# Optimizing differential identifiability improves connectome predictive modeling of cognitive deficits from functional connectivity in Alzheimer's disease

**DOI:** 10.1002/hbm.25448

**Published:** 2021-05-05

**Authors:** Diana O. Svaldi, Joaquín Goñi, Kausar Abbas, Enrico Amico, David G. Clark, Charanya Muralidharan, Mario Dzemidzic, John D. West, Shannon L. Risacher, Andrew J. Saykin, Liana G. Apostolova

**Affiliations:** ^1^ Indiana University School of Medicine Indianapolis Indiana USA; ^2^ School of Industrial Engineering Purdue University West Lafayette Indiana USA; ^3^ Purdue Institute for Integrative Neuroscience, Purdue University West Lafayette Indiana USA; ^4^ Weldon School of Biomedical Engineering Purdue University West Lafayette Indiana USA

**Keywords:** AD, Alzheimer's disease, cognition, fMRI, functional connectivity, functional fingerprinting, predictive modeling, resting state

## Abstract

Functional connectivity, as estimated using resting state functional MRI, has shown potential in bridging the gap between pathophysiology and cognition. However, clinical use of functional connectivity biomarkers is impeded by unreliable estimates of individual functional connectomes and lack of generalizability of models predicting cognitive outcomes from connectivity. To address these issues, we combine the frameworks of *connectome predictive modeling* and *differential identifiability*. Using the combined framework, we show that enhancing the individual fingerprint of resting state functional connectomes leads to robust identification of functional networks associated to cognitive outcomes and also improves prediction of cognitive outcomes from functional connectomes. Using a comprehensive spectrum of cognitive outcomes associated to Alzheimer's disease (AD), we identify and characterize functional networks associated to specific cognitive deficits exhibited in AD. This combined framework is an important step in making individual level predictions of cognition from resting state functional connectomes and in understanding the relationship between cognition and connectivity.

## Graphical abstract

Using differential identifiability and connectome predictive modeling, we show that enhancing the individual fingerprint of resting state functional connectomes leads to robust identification of functional networks associated to cognitive outcomes and also improves prediction of cognitive outcomes from functional connectomes. The framework allows us to more robustly identify and characterize functional networks associated to specific cognitive outcomes exhibited in AD.

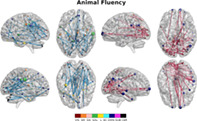

## 1 INTRODUCTION

The biological underpinnings of all neurodegenerative disorders remain poorly understood, contributing significantly to the bottleneck in treating these disorders (Montague, Dolan, Friston, & Dayan, 2012). In recent years, the application of analyses based on complex systems approaches for understanding how neural activity and connectivity facilitate cognition has led to significant strides in characterizing these disorders (Fornito & Bullmore, 2015; Fornito, Zalesky, & Breakspear, 2015). One such approach, functional brain connectomics, models functional brain networks as pairwise statistical dependencies in regional neural activity. This provides a framework to assess critical aspects of the brain, such as integration and segregation (Sporns, 2013), and ultimately communication (Avena‐Koenigsberger, Misic, & Sporns, 2017; Bullmore & Sporns, 2009). At the same time, the advent of functional MRI (fMRI) has allowed for in‐vivo characterization of whole brain functional connectomes (FC) in humans (Bullmore & Sporns, 2009), leading to the discovery of several critical brain networks implicated in schizophrenia, attention deficit hyperactivity disorder, autism, and Alzheimer's disease (AD) (Fornito & Bullmore, 2015).

Despite their potential to enhance our understanding of neurologic disorders, approaches based on functional connectivity have not yet been used translationally in the treatment of cognitive and behavioral disorders (Yahata, Kasai, & Kawato, 2017; Yamada et al., 2017). To advance the treatment of such disorders, there is a critical need to develop clinical biomarkers that are (a) robustly modulated by disease mechanisms and (b) specifically associated with disease related outcomes (Yamada et al., 2017). Though functional connectivity has shown potential in bridging the gap between pathophysiology and cognition, its clinical use is impeded by unreliable estimation of subject level FC (Braun et al., 2012), lack of precision in inter‐subject differentiability in FC (Noble et al., 2017), and lack of generalizability of models predicting subject‐level cognitive outcomes from FC (Yamada et al., 2017). Here we show that improving the subject level fingerprint of resting‐state FC also improves prediction of a heterogeneous set of cognitive deficits in AD, both in new data from the training cohort as well as data from a validation cohort. We also identify functional networks associated to specific cognitive deficits exhibited in AD.

### 1.1 Toward improving clinical utility of FC

While FC shows differential group level associations across cognitive outcomes (Amico, Arenas, & Goni, 2019; Amico & Goñi, 2018) and across disease conditions (Badhwar et al., 2017; Brier, Thomas, & Ances, 2014; Contreras et al., 2017; Fornito et al., 2015; Fornito & Bullmore, 2015; Svaldi et al., 2018), it falls short of predicting clinically meaningful outcomes at the individual level. The reason for this, is insufficient “fingerprint” or within‐subject reliability and between‐subject differentiability to capture individual differences that may be related to cognition or behavior (Amico & Goñi, 2018; Finn et al., 2015; Mars, Passingham, & Jbabdi, 2018; Pallares et al., 2018; Satterthwaite, Xia, & Bassett, 2018; Seitzman et al., 2019). In terms of reliability of FC, it has been shown that reasonable reliability can be achieved with sufficient scan length and that this reliability can be improved when multiple sessions of FC are used (Birn et al., 2013; Noble et al., 2017; Noble, Scheinost, & Constable, 2019). Studies have also shown that FC reliability is different across the brain, with larger cortical nodes displaying the most reliability and within network connections exhibiting greater reliability than between network connections (Noble et al., 2017). Several studies have also shown that frequently used 6 min fMRI acquisitions do not have adequate reliability at the edge level, posing significant issues on numerous available clinical fMRI datasets when performing subject level associations. In terms of inter‐subject differentiability, recent efforts have shown that individuals can be reasonably distinguished from each other using FC, as measured by identification rate (Amico & Goñi, 2018; Finn et al., 2015, 2017), perfect separability rate (Finn et al., 2015, 2017; Noble et al., 2017), or differential identifiability (Amico & Goñi, 2018). Furthermore, it has been shown that individual level fingerprinting improves with longer scan length (Amico & Goñi, 2018; Noble et al., 2017) and when subjects are performing specific tasks (Finn et al., 2017). Finally, there is evidence that FC fingerprint is reduced in individuals with neurologic or psychiatric conditions (Kaufmann et al., 2017, 2018; Svaldi et al., 2018), making association of FC with disease related phenotypes more difficult.

Evidence for fingerprint in FC has opened the door for efforts to improve prediction of cognition and behavior from FC (Scheinost et al., 2019; Shen et al., 2017; Yahata et al., 2017; Yamada et al., 2017; Yoo et al., 2018). Predictive pipelines typically involve: (a) feature reduction to find FC features that are associated with specific cognitive outcomes, (b) training of a predictive model using these features to predict cognitive outcomes, and (c) evaluation of the accuracy and generalizability of resulting models on external data. It has been demonstrated that using multiple connectomes across sessions or connectomes from different tasks improves predictive power (Gao, Greene, Constable, & Scheinost, 2019). However, how test/retest reliability of FC features affects their contribution to predicting cognition or behavior is still under debate. One study found no association between the test/retest reliability of a given functional edge and the predictive value of the edge in external data (Noble et al., 2017). However, another study (Amico & Goñi, 2018) showed increased prediction accuracy when edges were chosen based on correlation to cognitive outcome and test/retest reliability, after using PCA to optimize differential identifiability to uncover FC fingerprints.

Though strategies such as increased scan length, multiple acquisitions and adding task‐based fMRI may be useful for improving prediction of relevant outcomes, they may not be feasible in clinical settings. Reasons include the associated cost of increased scan time, diminished tolerance in patient populations to long scan times, and impaired ability of patients in performing tasks. Additionally, there are numerous available datasets (Abbas et al., 2015; Amico et al., 2017; Contreras et al., 2017; Petersen et al., 2010; Xiao et al., 2017) with acquisition protocols that do not have adequate reliability (Noble et al., 2017) for subject level prediction. Finally, prediction of cognitive outcomes in neurologic and psychiatric populations remains a challenge as these subjects appear to have a reduced fingerprint (Kaufmann et al., 2017, 2018). To address such issues, Amico and Goñi proposed the *differential identifiability* framework (If) (Amico & Goñi, 2018), which is a principal component analysis (PCA) based denoising algorithm to uncover fingerprints in FC and improve between‐subject differentiability at the same time. Using data from 100 unrelated subjects in the Human Connectome Project, they demonstrated improvements in FC fingerprint beyond what could be achieved by increasing scan length (Amico & Goñi, 2018). This improvement was also observed in FC data from the Alzheimer's Disease Neuroimaging Initiative (ADNI), a dataset with more traditional acquisition consisting of 140 volumes (7 min scan) split in half to mimic a test/retest setting (Svaldi et al., 2018). Thus, the If framework demonstrated improvements in fingerprinting in a “traditional” acquisition performed on a clinical population. However, there is still conflicting evidence on whether increasing FC fingerprint subsequently improves prediction of clinically relevant outcomes. It is important to note that the abovementioned studies specifically assessed whether the test/retest reliability of a functional edge affected the predictive value of that edge in external subjects. Hence, two important questions remain open (a) the level of agreement in feature selection between test and retest data from the same subjects and (b) whether a model built on test data would generalize to re‐test data from the same subjects. These two questions are critical, since good performance of predictive models in a test/retest setting is a minimum standard that should be met before testing on external data. Lack of agreement in feature selection between test and retest data indicates a model that overfits the training data and is not generalizable, even to a new session of the same training subjects. Even if this model is somewhat generalizable to external subjects, if it lacks test–retest agreement in feature selection, the model is likely overparametrized and selecting arbitrary features.

In this work, we test the effect of the differential If on key properties of models predicting cognitive outcomes related to AD from FC data. We assess performance of the models in both a test/retest setting and in generalization to validation data. When choosing key properties to assess the quality of predictive models for the purposes of predicting and understanding cognitive associations to the brain, it is important to keep in mind that interpretation of anatomical locations of the cognitive correlates of FC are as relevant as the accuracy of prediction. Hence, confirming robustness in the identification of FC features should precede model fitting and assessments of model accuracy. Further, it is important to note that the robustness of both feature selection and coefficient estimation can significantly influence model accuracy and generalizability. Therefore, we propose to evaluate three critical properties for *well‐behaved* FC‐based predictive models: (a) stability of feature selection in a test/retest setting, (b) specificity of edge selection, and (c) generalizability of the prediction to new data from the same subjects and to validation data.

### 1.2 Opportunities in AD

We chose to evaluate these effects in data from the ADNI2, which consists of subjects spanning the AD spectrum. The heterogeneous, gradual progression of cognitive deficits in AD is particularly amenable to study the quality of models predicting cognition from FC. Briefly, in the stage of mild cognitive impairment (MCI) subjects typically manifest episodic memory decline, which is later accompanied by subtle deficits in other domains, and ultimately results in progressive functional impairment as the subject transitions through the mild, moderate and severe stages of dementia (Aggarwal, Wilson, Beck, Bienias, & Bennett, 2005; Cloutier, Chertkow, Kergoat, Gauthier, & Belleville, 2015; Lambon Ralph, Patterson, Graham, Dawson, & Hodges, 2003; Zhao et al., 2014). Within the AD spectrum there is much individual heterogeneity in terms of disease presentation and progression over time (Lambon Ralph et al., 2003), making predictive modeling at the subject level important.

The association between FC changes and cognitive deficits in AD has been subject of intense study to date (Contreras et al., 2017; Wook Yoo et al., 2015; Zhan et al., 2016). Changes in functional networks, primarily the default mode and frontoparietal networks, have been consistently replicated between diagnostic groups (Buckner et al., 2005, 2009; Seeley, Crawford, Zhou, Miller, & Greicius, 2009). Recent studies indicate that FC data can predict subject level diagnostic status (Vogel et al., 2018) and global cognitive decline (Lin et al., 2018) with reasonable accuracy. Several studies also show relationships between FC data and deficits in specific cognitive domains associated with AD (Contreras et al., 2017; Duchek et al., 2013; Zhan et al., 2016).

In this work, beyond assessing group level associations to specific cognitive domains or individual level prediction of cognitive status (impaired vs. non‐impaired), we present a framework that improves the ability of FC to predict subject level deficits from different cognitive domains. This additionally enables us to assess which RSNs are globally associated to cognition in AD versus RSNs associated to specific deficits observed in AD.

## 2 METHODS

### 2.1 Subject demographics and cognitive performance

Data used in the preparation of this article were obtained from the ADNI database (adni.loni.usc.edu). The ADNI was launched in 2003 as a public‐private partnership, led by Principal Investigator Michael W. Weiner, MD. The primary goal of ADNI has been to test whether serial magnetic resonance imaging (MRI), positron emission tomography (PET), other biological markers, and clinical and neuropsychological assessment can be combined to measure the progression of MCI and AD. For up‐to‐date information, see www.adni-info.org.

In this work, resting state fMRI and neurocognitive testing data from the second phase of the ADNI2/GO were used. Our analyses included 82 participants from the original 164 ADNI2/GO individuals with resting state fMRI scans. Subjects were excluded if (a) their amyloid status was not available, (b) they were cognitively impaired, but showed no evidence of amyloid‐beta (Aß) deposition, and/or (c) they had over 30% of fMRI time points censored due to artifacts or head motion (see Section 2.2 for details). Aß status was determined using either mean PET Florbetapir standard uptake value ratio cutoff (Florbetapir >1.1; UC Berkeley) or CSF Aß level ≤ 192 pg/ml [5]. The rationale for excluding Aß‐cognitively impaired participants was to ensure that all impaired subjects had underlying AD pathology, in an attempt to keep the pathological substrates of cognitive impairment as homogenous as possible in the sample. Subjects were stratified into five categories based on their diagnosis and Aß status (Table 1): (a) Aß‐cognitively normal individuals (CN_Aß−,_ *n* = 15), (b) Aß + CN or pre‐clinical AD (CN_Aß+_, *n* = 12), (c) early MCI due to AD (EMCI_Aß+_, *n* = 22), (d) late MCI due to AD (LMCI_Aß+_, *n* = 12), and (e) AD dementia (AD_Aß+_, *n* = 21).

**TABLE 1:** Demographics and neurocognitive comparisons of diagnostic groups

Variable mean (*SD*)	CN_Aß‐_ (*n* = 15)	CN_Aß+_ (*n* = 12)	EMCI_Aß+_ (*n* = 22)	LMCI_Aß+_ (*n* = 12)	AD_Aß+_ (*n* = 21)
Age (years)	74.2 (8.8)	75.9 (7.0)	72.6 (5.2)	73.3 (6.1)	73.5 (7.6)
Sex (% F)	64.2	41.7	50	61.6	42.9
Years of education	16.7 (2.3)	15.8 (2.6)	15.2 (2.6)	16 (1.8)	15.4 (2.6)
MOCA^a^	26.2 (2.6)	25.3 (2.9)	22.3 (4.5)	20.6 (7.1)	13.4 (5.2)
Auditory verbal learning Immediate recall^a^	11.1 (3.0)	11.33 (2.9)	9.9 (3.0)	7.6 (2.4)	4.3 (1.6)
Auditory verbal learning Delayed recall^a^	6.2 (4.3)	7.8 (3.8)	4.3 (4.0)	2.8 (2.8)	0.4 (0.9)
Boston naming^a^	28.2 (2.0)	28.7 (1.1)	27.1 (3.1)	25.9 (5.0)	22.4 (6.4)
Animal fluency^a^	21.1 (3.64)	20.1 (3.6)	18.8 (4.2)	17.4 (4.8)	12.3 (5.0)
Clock drawing^a^	4.8 (0.4)	4.5 (1.0)	4.6 (0.5)	3.8 (1.3)	3.1 (1.3)
Trail making B^a^	69.0 (22.6)	81.4 (19.6)	99.9 (43.1)	131 (89.0)	216.9 (75.6)

Abbreviations: AD, Alzheimer's disease; CN, cognitively normal; EMCI, early mild cognitive impairment; LMCI, late mild cognitive impairment; MOCA, Montreal cognitive assessment.

^a^ Significant group effect (Chi‐squared or analysis of variance as appropriate, *α* = .05). Values in parenthesis denote *SD*.

We used five outcome measures for predictive modeling from the ADNI2/GO neurocognitive battery which exhibited a significant effect of diagnosis (analysis of variance, *α* = .05) in the 82 subjects and spanned the cognitive spectrum (www.adni-info.org for protocols): the auditory verbal learning test (AVLT) immediate recall, AVLT delayed recall, clock drawing, Trail Making B, Animal Fluency. Additionally, the Montreal cognitive assessment (MOCA) was also included as a representative clinical measure of global cognition. Of note, all outcome measures were z‐scored, relative to the training data, prior to predictive modeling to allow for direct comparison between models across outcome measures.

### 2.2 fMRI data processing

We used T1‐weighted MPRAGE scans and EPI fMRI scans from the initial visit in ADNI2/GO (Philips Platforms, TR/TE = 3000/30 ms, 140 volumes, 3.3 mm thickness, see www.adni-info.org for detailed protocols) for estimation of whole‐brain FCs. fMRI scans were processed with an in‐house MATLAB and FSL based pipeline (Amico et al., 2017). This pipeline follows previously proposed processing guidelines (J. D. Power, Barnes, Snyder, Schlaggar, & Petersen, 2012; J. D. Power et al., 2014). For purposes of evaluating reproducibility, we split the processed fMRI time series into halves (mimicking test and retest) and assigned each half for each subject as “restA” or “restB” randomly to avoid biases related to first versus second half of the scan.

We obtained two FC matrices from the restA and restB halves of the fMRI time‐series for each subject. FC nodes were defined using a 278 region parcellation (Shen, Tokoglu, Papademetris, & Constable, 2013), as previously detailed (Amico et al., 2017), with a modified more fine grained subcortical parcellation (Mawlawi et al., 2001), for a total of 286 Gy matter regions. We estimated single session FC matrices by calculating the Pearson correlation coefficient (K. Pearson, 1901) between the fMRI time‐series of each pair of brain regions. Each region's time‐series was obtained by averaging time‐series of all voxels assigned to that brain region. Regions were assigned to one of the seven cortical resting state subnetworks (RSN/RSNs): visual (VIS), somato‐motor (SM), dorsal attention (DA), salience (SAL), limbic (L), executive control (EC), and default mode network (DMN) (Yeo, Krienen, Chee, & Buckner, 2014), with the remaining regions assigned to a subcortical (SUB) or cerebellar (CER) networks.

### 2.3 Differential If

**FIGURE 1:**
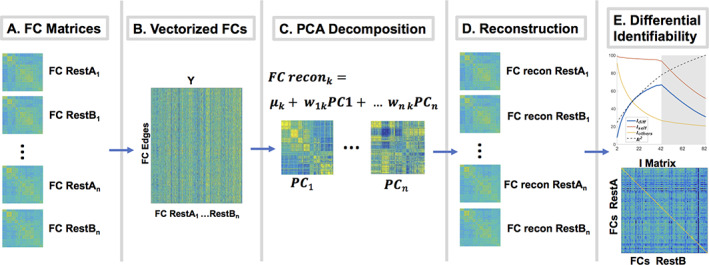
Differential identifiability framework (If). (a) For each subject, two functional connectomes (FC) matrices (restA and restB) were estimated for each half of the fMRI time‐series. (b) FC matrices were vectorized (upper triangular) and placed into a group FC matrix. (c) Principal component analysis (PCA) decomposition was performed on the group FC matrix. Each PC can be arranged as a matrix in the FC domain. (d) Individual FCs were reconstructed using different number of PCs. (e) *I*_diff_ was estimated for different number of PCs (in order of explained variance) and the number of PCs maximizing *I*_diff_ found

We applied If which uses group level PCA (Hotelling, 1933) to find the optimal FC reconstruction point for simultaneous optimization of restA and restB FC reproducibility and between‐subject differentiability, measured using differential identifiability (*I*_diff_, Figure 1) (Amico & Goñi, 2018). This section describes the general steps in the framework, while the cohorts on which the data was applied are described in Section 2.4. In this framework, the “identifiability matrix” ***I*** is defined as the matrix of pairwise correlations (square, non‐symmetric) between the subjects' *FC*_restA_ and *FC*_restB_. The dimension of ***I*** is *N* ^2^ where *N* is the number of subjects in the cohort. Self‐identifiability, (*I*_self_, Equation 1), is defined as the average of the main diagonal elements of ***I***, consisting of correlations between *FC*_restA_ and *FC*_restB_ from the same subjects. *I*_others_ (Equation 2), is defined as average of the off‐diagonal elements of matrix ***I***, consisting of correlations between *FC*_restA_ and *FC*_restB_ of different subjects. Differential identifiability (*I*_diff_, Equation 3) is defined as the difference between *I*_self_ and I_others_.

(1) $$I_{\text{self}}=\frac{1}{N}\sum_{i=1}^{N}I_{i,i}$$

(2) $$I_{\text{others}}=\frac{1}{2\left(\begin{array}{c} N\\ 2 \end{array}\right)}\sum_{i\neq j}I_{i,j}$$

(3) $$I_{\text{diff}}=100\times \left(I_{\text{self}}-I_{\text{others}}\right)\;$$

Group level PCA is then applied in the FC domain, on a data matrix (Figure 1a,b) containing vectorized *FC*_restA_ and *FC*_restB_ (upper triangular of FC matrices excluding main diagonal) from all subjects in a given cohort (see Section 2.4 for information on cohorts). Following PCA decomposition (Figure 1c,d) all FCs in the cohort are iteratively reconstructed and *I*_diff_ is quantified for a range of number of PCs (Figure 1e). Optimal FC matrices are reconstructed using the number of PCs optimizing *I*_diff_. Following implementation of the If framework, fingerprint at the functional edgewise level for each subject is evaluated for the original FC matrices (matrices reconstructed suing the full range of PCs) versus optimally reconstructed FC matrices using the intraclass correlation coefficient (ICC 2,1) (Shrout & Fleiss, 1979).

### 2.4 Connectome predictive modeling and cross validation scheme

**FIGURE 2:**
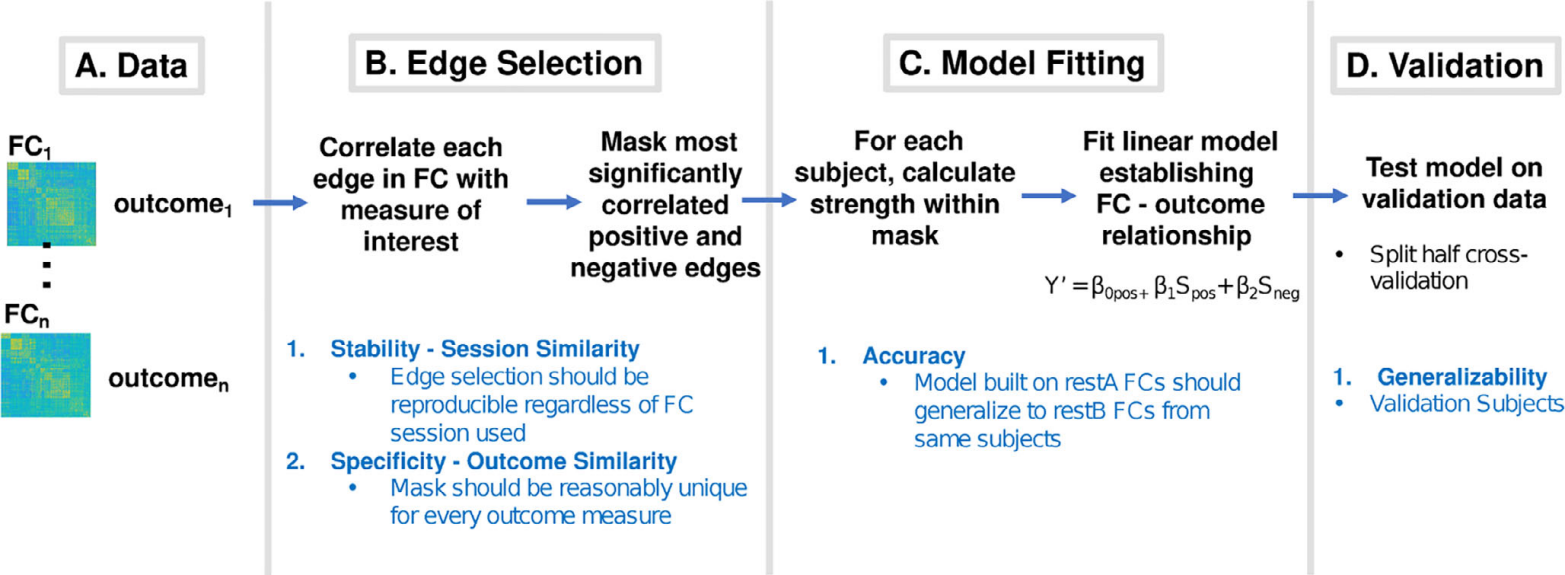
Connectome predictive modeling scheme (adapted from Shen et al., 2017). Black text delineates procedures for each step while blue text delineates properties that are important at each step to achieve an overall robust model. (a) The goal is to predict the outcome measure from functional connectomes (FC) data. (b) Edgewise correlations were performed with outcome of interest. Most significantly positively and negatively correlated edges were selected. Here stability of edge selection regardless of restA versus restB FC data used is important. (c) Strength in the positive and negative restA masks were computed using restA FCs. Strengths were used as regressors in a linear model predicting the outcome measure. Here is important that the resulting model generalize to restB data from the same subjects. (d) Model generalizability to validation data was assessed. Here, it is important that the final model is generalizable to external data

The connectome predictive modeling pipeline (CPM) (Shen et al., 2017) was used to assess the effect of If (Amico & Goñi, 2018) on predictive modeling of the aforementioned outcome measures. Briefly, the pipeline consists of three steps (Figure 2). First, *edge selection* (Figure 2b) is performed by computing the correlation between each edge (from a total of 40,755 edges) and each outcome measure. Edges exhibiting an absolute value of correlation above a certain threshold (threshold = 0.1 here) are selected to create a positive correlation mask and negative correlation mask. Second, the *model fitting* (Figure 2c) portion of CPM is performed. To estimate a model for each outcome measure, strength (sum of all edges in the mask) in the positive and negative masks are used as predictors in a linear regression model. Third, *model validation* is performed on external data, typically using a k‐fold cross validation scheme.

**FIGURE 3:**
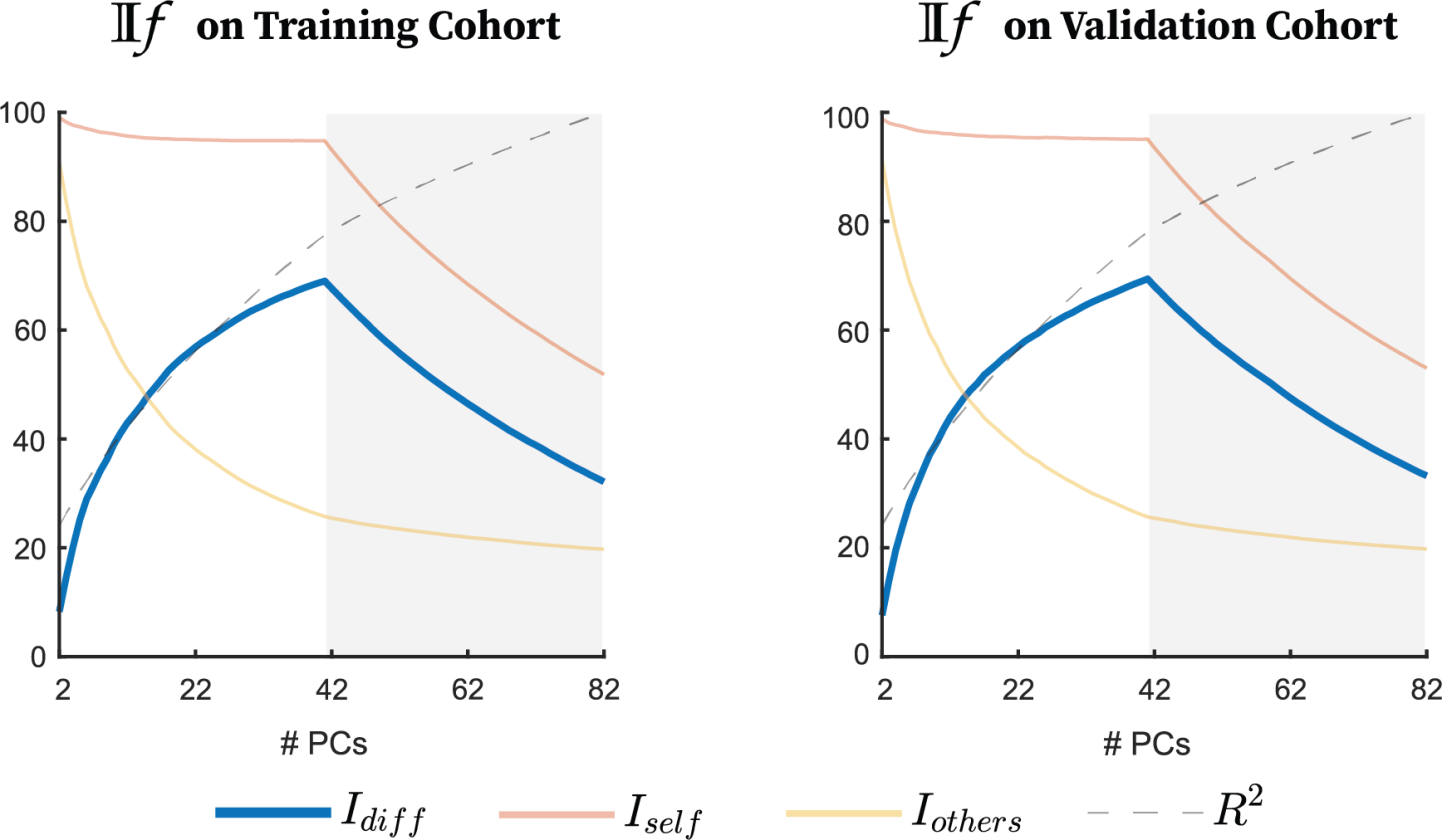
Mean of identifiability framework (If) assessments on training cohort (left) and Testing cohort (right). Connectome level identifiability assessment. *I*_self_ and *I*_others_ represent similarity between test and retest functional connectomes (FCs) of the same versus different subjects, respectively, across number of PCs used for reconstruction. Differential identifiability (*I*_diff_) is the difference between *I*_self_ and *I*_others_. The cumulative percent explained variance (100 × *R* ^2^) across number of PCs used for reconstruction is also included

For this work, the entire cohort (*N* = 82) was split into a training cohort (*N* = 41) and validation cohort (*N* = 41) in a split half, cross validation scheme (1,000 repetitions). This was chosen because split half cross validation has shown to have the least amount of variance in performance across repetitions, for a constant training size (Scheinost et al., 2019). In each repetition, 41 subjects were randomly selected as training subjects and the other 41 were selected as validation subjects. If was performed separately on matrices containing restA and restB FCs from training versus validation cohorts. Average *I*_self_, *I*_others_, *I*_diff_, *R* ^2^ (Figure 3) plus ICC (Figure S1), across the 1,000 repetitions, are reported for the training versus validation cohorts. Model estimation (one per cognitive outcome) was performed using restA FCs of the training cohort. The resulting CPM models were then evaluated on restB FCs of the training cohort (see Section 2.5 paragraph 1–2). Finally, an external evaluation of the performance of these models was carried out using the validation cohort. Such evaluation was done by comparing the performance of models built/tested on original FCs (reconstructed using the full range of PCs) to the performance of models built/tested on FCs optimally reconstructed for *I*_diff_ (see Section 2.5 last paragraph).

### 2.5 Assessment of differential identifiability pipeline on connectome predictive modeling

**FIGURE 4:**
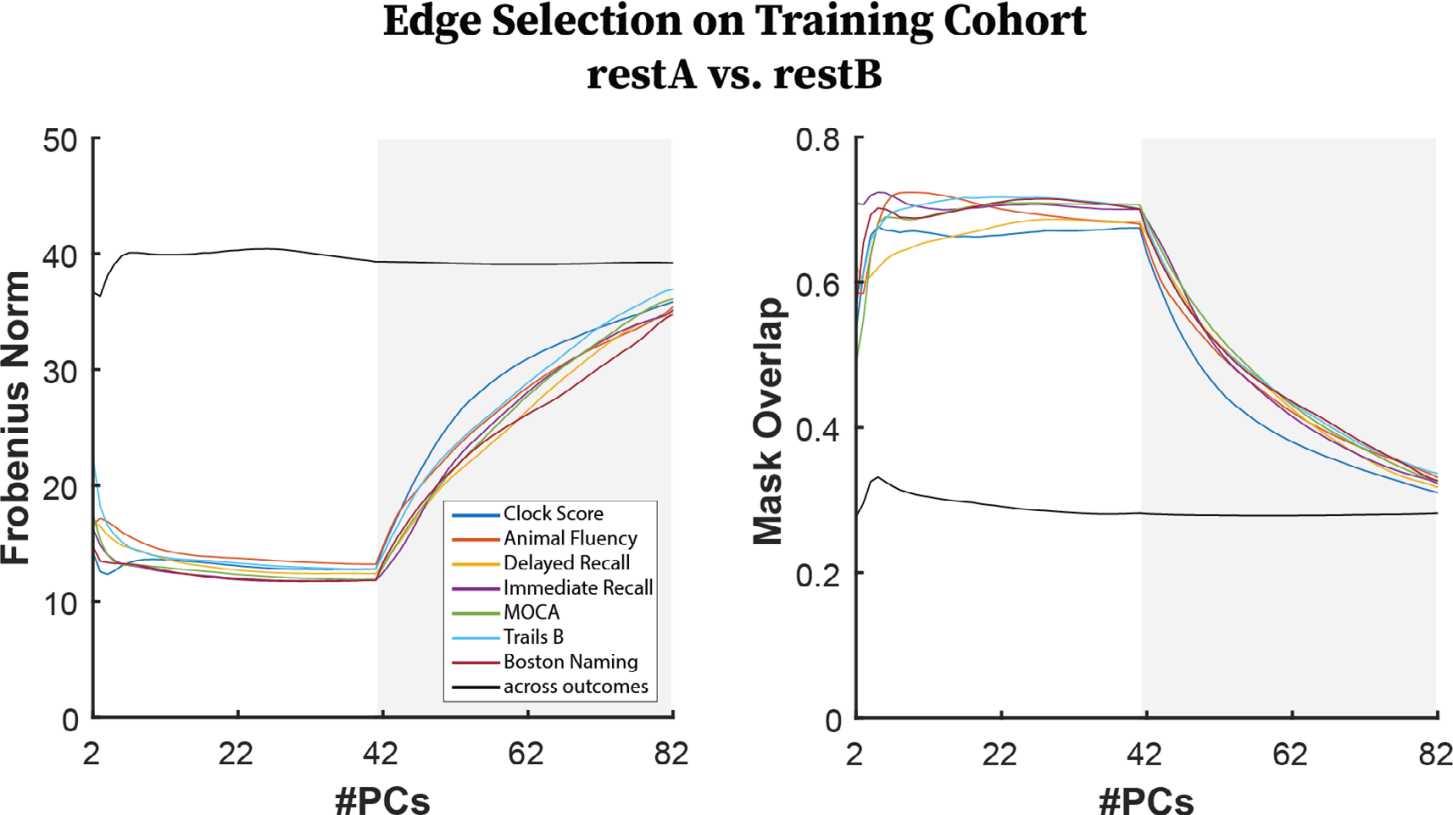
(Left) (Colored Lines) Frobenius norm of correlation matrices associated to each outcome measure for restA functional connectomes (FCs) versus restB FCs. (Black line) Average pairwise Frobenius Norm of correlation matrices between two different outcome measures using only restA FCs. (Right) (Colored Lines) Mask overlap between restA FCs versus RestB FCs, for each outcome measure. (Black Line) Average pairwise mask overlap between two different outcomes using only restA FCs

We first evaluated the stability and specificity of the predictive modeling pipeline in a test–retest setting on the training subjects by performing edge selection separately on restA versus restB FCs (Figure 4). *Stability in FC‐outcome correlation*: For each repetition, we evaluated stability in edgewise correlation using the Frobenius norm between restA and restB correlations, where values close to zero denote high similarity between restA and restB correlation vectors. *Stability in edge selection*: We evaluated the similarity of overlap in selected edges as the number of edges selected (same sign) using both restA and restB FCs divided by the number of edges selected using either restA or restB FCs. *Specificity of edge selection*: Additionally, we evaluated specificity of edge selection by calculating average, pairwise Frobenius norms and mask overlaps across all outcome measures using only restA FCs. Averaged Frobenius norm and percent overlap over the 1,000 repetitions was reported for both stability and specificity across the range of PCs.

**FIGURE 5:**
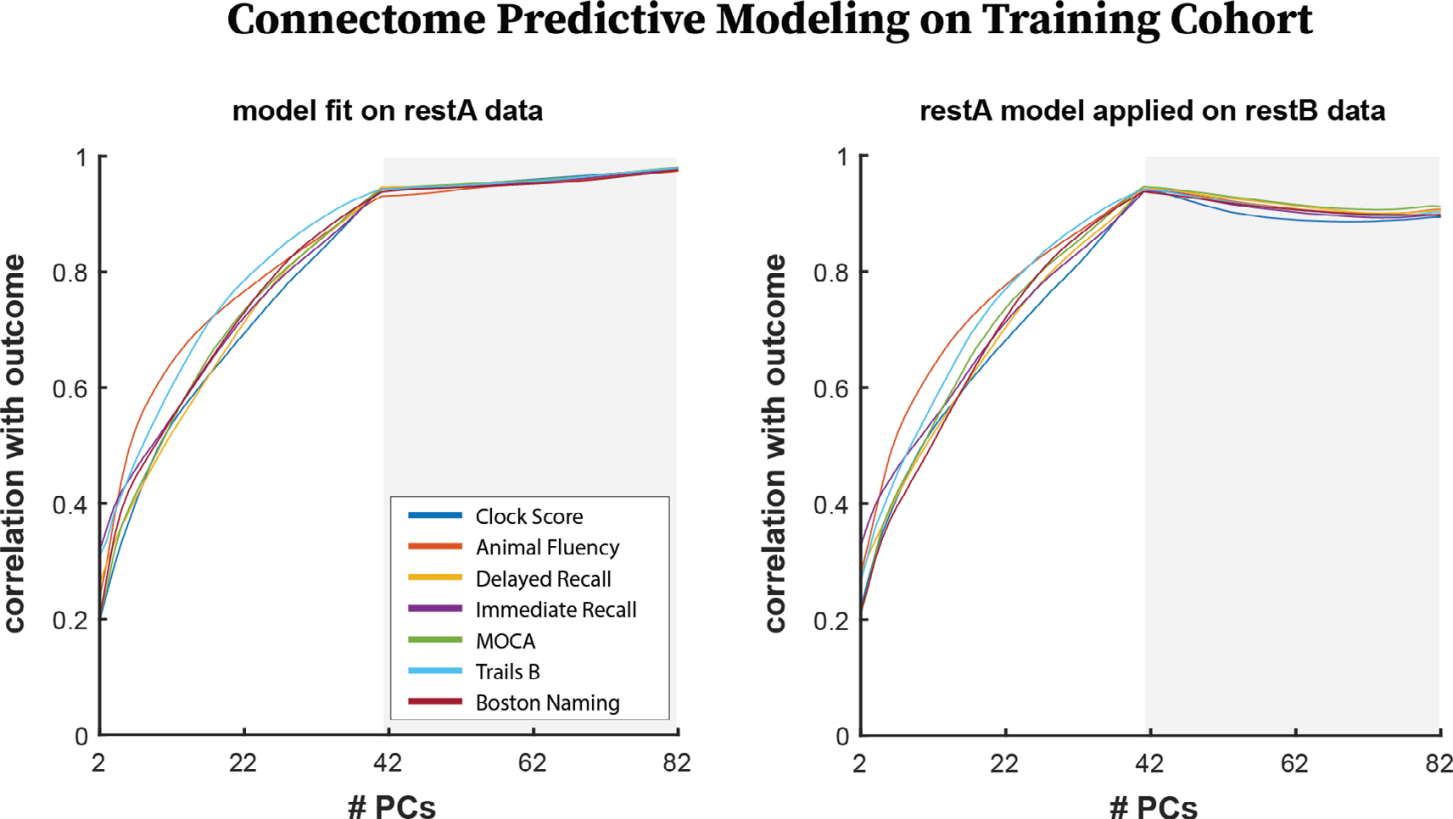
For all plots, restA Training functional connectomes (FCs) were used for edge selection and model fitting. (left) Correlation between estimated and expected outcomes from models fit using restA FCs. (right) Correlation between estimated and expected outcomes when models fit on restA Training FCs were applied to restB FCs from the same subjects

We also evaluated the model fitting portion of the CPM pipeline in a test–retest setting by performing model fitting on restA data and subsequently evaluating the resulting model on restB data (Figure 5). Pearson correlation of estimated versus observed outcomes were used to evaluate model generalizability from restA to restB data in training subjects. Average Pearson correlation over the 1,000 repetitions are reported for restA FCs (Figure 5 left) and restB FCs (Figure 5 right) reconstructed across the range of PCs.

**FIGURE 6:**
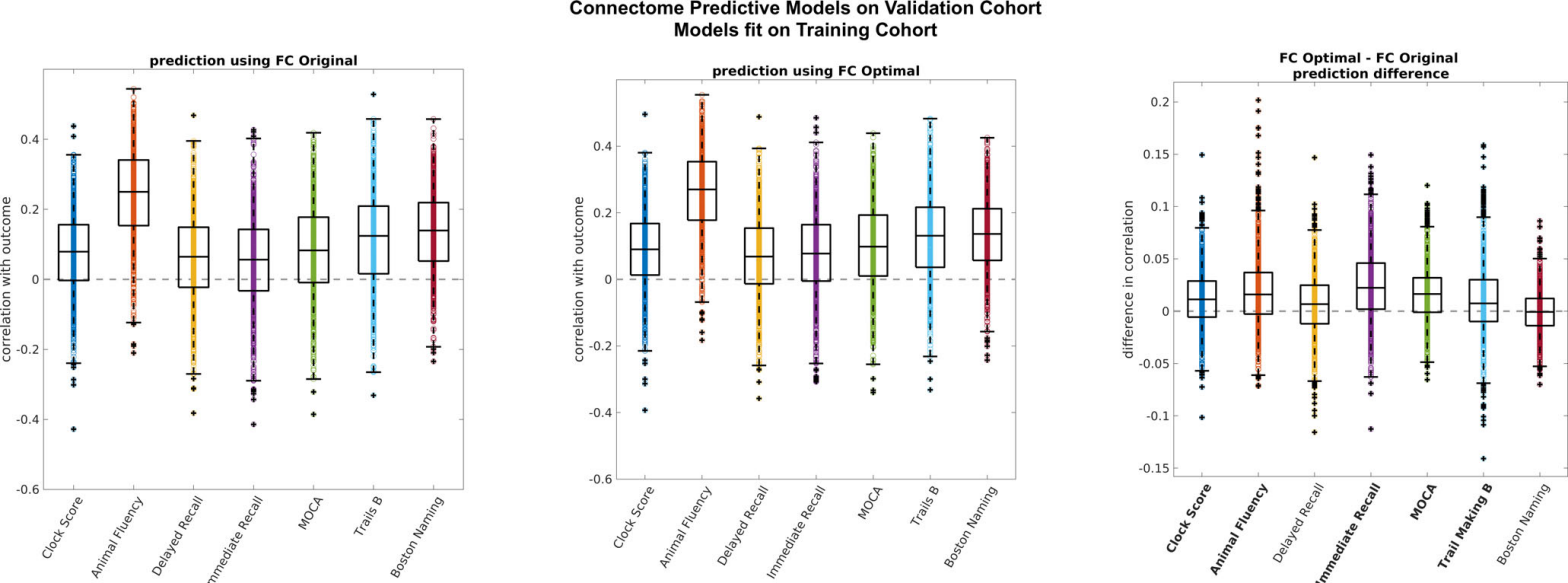
Model performance in validation cohort across 1,000 repetitions. Asterisk indicates outcomes for which performance was significantly improved in optimally reconstructed functional connectomes (FCs) versus original FCs (non‐parametric permutation test, *α* = .01 corrected using *t*_max_ method). The center line of each box corresponds to the median and the bounds to the 25th and 75th percentiles. Outliers are defined using 1.5*inter quartile range. (left) Correlation between estimated and expected outcomes in original FCs from the validation cohort. Models were fit using original FCs from the training cohort. (middle) Correlation between estimated and expected outcomes in optimally reconstructed FCs from the validation cohort. Models were fit on optimally reconstructed FCs from the training cohort. (right) Difference in correlation between optimally reconstructed FCs and original FCs

Finally, we compared performance of the models estimated from training subjects on the FCs of validation subjects (Figure 6). Here it is important to note that the optimal restA‐restB averaged FCs share an average correlation of .99 with the original restA‐restB averaged FCs. However, the optimally reconstructed restA and restB FCs are much more correlated to each other (Figure 3), and individually more correlated to their average (.99 average correlation) than are the original restA and restB FCs (.87 average correlation). This means that by performing If, we obtain two good quality FCs where we previously obtained one. Because of this, for original FCs, we ran edge selection on the restA‐restB averaged FC and applied the masks and the model coefficients from the average restA‐restB FC matrix of training subjects to the restA‐restB average FC matrices of validation subjects. However, for optimal FCs, we ran edge selection separately on restA versus restB FCs and took the intersect mask, representing those edges which were robust across both masks. We then used this mask to estimate two separate models using restA FCs versus restB FCs. Finally, we averaged the coefficients from these two models before applying the model and masks to the testing data. The model from the training data was applied separately to restA and restB sessions from the testing data and the two predictions were averaged. Pearson correlation was again used to evaluate performance of the model on the validation subjects. Averaged Pearson correlation over the 1,000 repetitions is reported for original FCs and for optimal FCs. To compare performance of models built/tested on original FCs versus models built/tested on optimally reconstructed FCs, non‐parametric paired permutation tests (*α* = .01, 1,000 permutations, corrected using *t*_max_ method) (Blair & Karniski, 1993), using original FCs versus optimal FCs at each repetition as paired samples, were conducted on the Pearson correlations.

### 2.6 Effect of pre‐processing on If‐CPM workflow

As a supplementary analysis, we split the timeseries prior to pre‐processing for evaluation of the effect of splitting data prior to versus after pre‐processing on the If‐CPM workflow. We were not able to implement the splitting of the time series directly in half prior to pre‐processing as such change greatly compromised the resulting FC, as expected, due to the short scanning length (7 min). Thus, we followed previously proposed guidelines (Horien et al., 2018) and split the time series by taking interleaved time points (as if TR = 6 s). We then repeated the entire scheme described above on connectomes generated from time series split prior to pre‐processing. As such we generated If‐CPM workflow results using two different approaches: **(1)** by processing the entire timeseries then splitting the pre‐processed time series in half and **(2)** by splitting the timeseries in an interleaved fashion then pre‐processing the split time series separately.

To test the effect of pre‐processing on the FC matrices, we performed pairwise correlations on restA‐restB averaged matrices from **(1)** versus **(2)** (Figure S9a). This allowed us to compare how similar restA and restB connectomes when generated using **(1)** versus **(2)**. We also performed a permutation test (*α* = .05, 1,000 permutations) on the *I*_*self*_ values in the main diagonal of the original ***I*** matrices (average across 1,000 repetitions) generated for **(1)** versus **(2)**. This allowed us to test whether splitting the data prior to pre‐processing versus splitting the data after pre‐processing affected the initial similarity between restA FCs and restB FCs from the same subjects (Figure 3 vs. S9b). To elaborate on the effect of pre‐processing on the entire If‐CPM workflow, we report qualitative comparisons of the behavior across the range of PCs of If, edge selection, and model generalizability in CPM for **(1)** versus **(2)** (Figures 4, 5, 6 vs. S9c–f).

### 2.7 Association of resting‐state networks to cognitive outcomes

**FIGURE 7:**
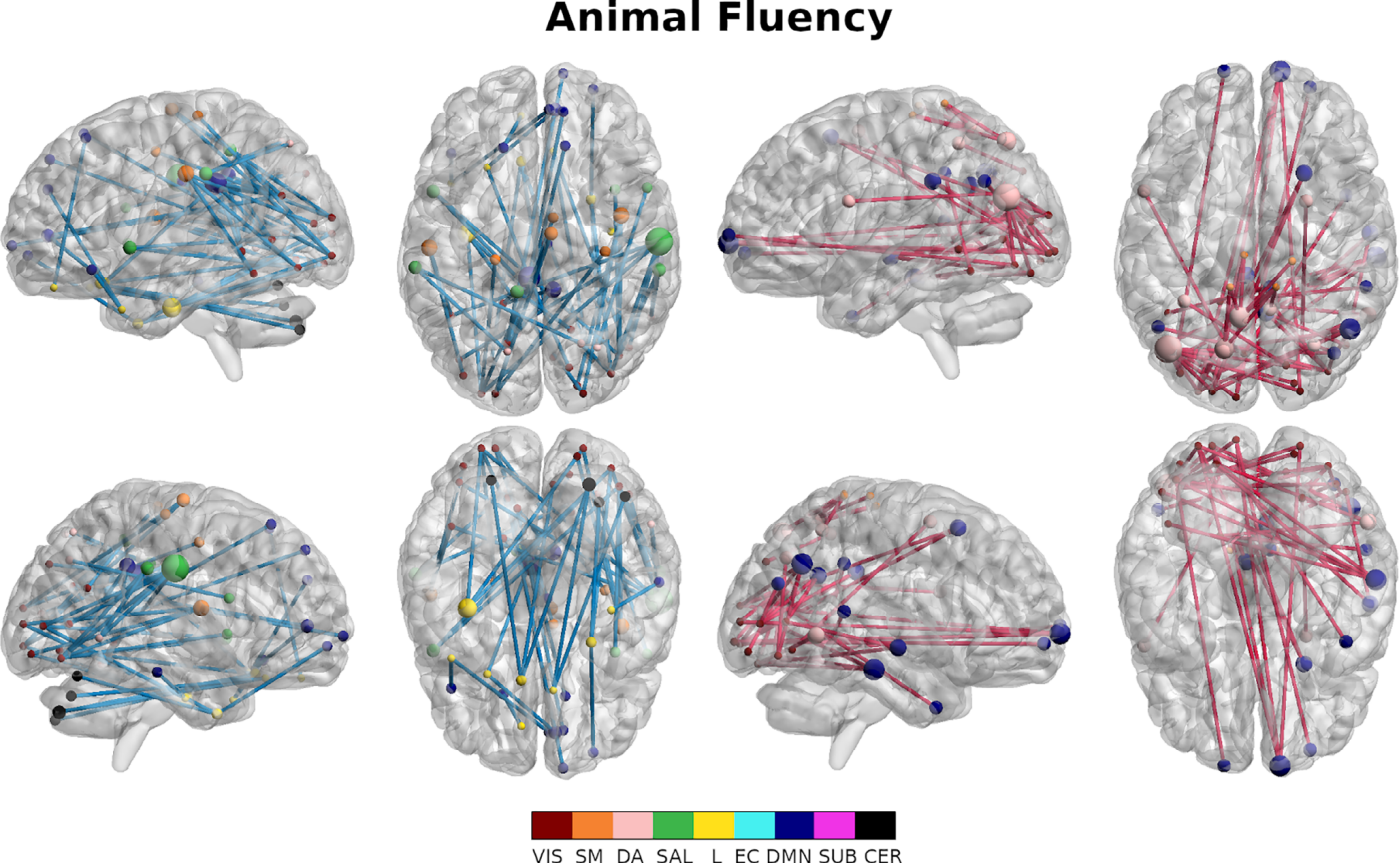
Overrepresented edges (binomial test, *α* = .01) for the animal fluency test. Positively associated edges (left) and negatively associated edges (right) are visualized separately. Nodes are sized according to their degree and colored according to resting state network membership. Positive mask edges are colored blue while negative mask edges are colored red

Final masks for each outcome measure were defined by edges that appeared in at least 95% of the 1,000 repetitions. We used binomial tests (*α* = .05, uncorrected) for each outcome measure to assess whether specific RSNs (e.g., DMN‐DMN), or their interactions (e.g., DMN‐FP), were overrepresented in these masks beyond what could be expected from an equal number of edges chosen at random. Edges from overrepresented networks (or interactions) were visualized using BrainNet viewer (Figures 7 and [Link], [Link]) (Xia, Wang, & He, 2013).

## 3 RESULTS

### 3.1 Differential identifiability

For both training and testing cohorts, *I*_diff_ peaked at 41 PCs regardless of repetition (Figure 3, mean training *I*_diff_ = 67.09 and mean validation *I*_diff_ = 68.77, mean training *I*_self_ = 81.62 and mean validation *I*_self_ = 81.68, mean training *I*_others_ = 32.46 and mean validation *I*_others_ = 33.01, mean training % variance explained = 71.86, mean validation % variance explained = 71.80). We observed an almost twofold increase in differential identifiability in the optimally reconstructed data (Figures 3 and S1). Such increase in whole‐brain differential identifiability also increased the fingerprint at the functional edge level, as shown when using mean ICC (Figure S2).

### 3.2 Edge selection—Stability and specificity

Stability in edge selection between restA and restB (Figure 4, colored lines) exhibited an optimal and stable range between 10 and 41 PCs both in terms of correlations and resulting selected edges associated to each outcome. The Frobenius norm of the edgewise correlation associated to each outcome measure (Figure 4 left) exhibited stable range of minimal divergence between RestA and RestB (10–41 PCs) after which divergence began to monotonically increase for all outcome measures. Overlap (Figure 4 right) between edges selected in RestA and RestB exhibited an optimal range of overlap (68% Clock Score–76% Trail Making B) in the range of 10–41 PCs, then monotonically decreased after 41 PCs for all outcome measures. Specificity of edge selection, measured as pairwise similarity across outcomes (Figure 4, black lines), was stable across PCs for both the Frobenius norm and mask overlap. It is critical to highlight that when all PCs were used for reconstruction (i.e., using original FC data) restA‐restB similarity (colored lines) approached similarity across outcomes of restA masks (black line).

### 3.3 Training data: Test–retest generalizability

At the model fitting step, the performance of models built on restA data was evaluated on restA and restB FCs from the same subjects. For restA connectomes, on which the models were built, correlation between the predicted and estimated outcomes increased as the number of PCs increased, though at a slower rate after the optimal reconstruction point for *I*_diff_ (Figure 5 left). In contrast, correlation between the predicted and estimated outcomes peaked at the optimal reconstruction point for *I*_diff_ in the training data (41 PCs) when the model was applied on restB connectomes (Figure 5 right) and slightly decayed after 41 PCs.

### 3.4 Validation cohort: generalizability

Model performance was measured as mean correlation between the estimated and predicted outcomes across repetitions. Model performance was compared between original FCs and optimally reconstructed FCs. Model performance from original FCs from the validation cohort ranged from 0.05 (±0.13) to 0.24 (±0.13) across outcomes (Figure 6 left). Model performance from optimally reconstructed FCs from the validation cohort ranged from 0.07 (±0.12) to 0.26 (±0.13) across outcomes (Figure 6 middle). Model performance on validation data was significantly higher for optimally reconstructed FCs versus original FCs in 5 of 7 outcomes (paired permutation test, *α* = .01, 1,000 permutations) (Figure 6 right).

### 3.5 Effect of pre‐processing on If‐CPM workflow

FCs were generated **(1)** by processing the entire timeseries together, then splitting the pre‐processed time series in half and **(2)** by splitting the timeseries in an interleaved fashion (see Section 2.6), then pre‐processing the split time series separately. The average pair‐wise correlation between FCs generated by **(1)** versus **(2)** was .77. *I*_self_ values, which measures correlation of restA FCs and restB FCs produced using the same method, were not significantly different between the two methods (Figure 3 vs. S9b). Stability and specificity in edge selection behaved similarly for the two methods (Figure 4 vs. S9c). Similarly, generalizability of restA models to restB models behaved similarly for the two methods. (Figure 5 vs. S9d). Finally, when fMRI series were split prior to pre‐processing, optimally reconstructed FCs generalized significantly better than original FCs to the validation cohort in 4/7 outcomes.

### 3.6 Association of resting state networks to cognitive outcomes

We identified RSN interactions playing significant roles in prediction of each cognitive outcome and then assessed patterns in RSNs involved across cognitive outcomes (Table 2). Interactions involving the DMN and the VA networks were most common (19 selection); followed by the VIS network (16 selections); the SM, EC, and CER networks (15 selections); the SAL and SUB networks (14 selections), and finally the L network (9 selections). Within network connections had a significant role in five masks, while between network connections played a significant role in all masks. A total of 34 out of 45 possible RSN interactions were significantly over‐represented, in either the positive or negative mask, across outcomes. RSN interactions that were overrepresented in the prediction of MOCA included DA‐VA, DA‐L, L‐CER and SUB‐CER in the positive mask for MOCA (Figure S3 left) and SM‐L, SM‐SUB, DA‐L, DA‐CER, SAL‐EC, and EC‐EC interactions were significantly over represented in the negative mask (Figure S3 right). For AVLT immediate recall, VIS‐SM, VIS‐SAL, and SUB‐CER interactions were significantly over represented in the positive mask (Figure S4 left) and SM‐DA, SM‐SUB, SAL‐EC, and SAL‐DMN were significantly over represented in the negative mask (Figure S4 right). For AVLT delayed recall, VIS‐SM, VIS‐DMN, L‐CER, SAL‐CER, EC‐CER, and DMN‐CER were over represented in the positive mask (Figure S5 left) and SAL‐DMN, DMN‐DMN, and SUB‐CER were significantly over represented in the negative mask (Figure S5 right). For Boston Naming, VIS‐VIS, VIS‐SAL, SM‐DA, DA‐SAL, DA‐CER, L‐DMN, EC‐SUB, and DMN‐SUB were over represented in the positive mask (Figure S6 left) and VIS‐DA, VIS‐DMN, SM‐SUB, SM‐CER, DA‐DMN, and EC‐DMN were over represented in the negative mask (Figure S6 right). For animal fluency, VIS‐SM, VIS‐SAL, VIS‐L, VIS‐DMN, DA‐SAL, L‐DMN, and L‐CER were significantly over represented in the positive mask (Figure 7 left), while VIS‐DA, VIS‐DMN, and SM‐DA were significantly over represented in the negative mask (Figure 7 right). For clock drawing, SM‐EC, and EC‐SUB were significantly over represented in the positive mask (Figure S7 left), while VIS‐SUB, SM‐SUB, DA‐SUB and EC‐DMN were significantly over represented in the negative mask (Figure S7 right). Finally for Trail Making B, VIS‐DA, SM‐SUB, DA‐CER, SAL‐EC, EC‐EC, EC‐DMN, and DMN‐CER were over represented in the positive mask (Figure S8 left), while SM‐SAL, DA‐DA, DA‐EC, DMN‐DMN, and CER‐CER were significantly over represented in the negative mask (Figure S8 right).

**TABLE 2:** Significantly overrepresented resting state networks for each outcome measure

Significant resting state networks		
Outcome measure	Positive mask	Negative mask
MOCA	DA‐VA DA‐L L‐CER SUB‐CER	SM‐L SM‐SUB DA‐L DA‐CER SAL‐EC EC‐EC
Auditory learning immediate recall	VIS‐SM SAL‐SAL SUB‐CER	SM‐DA SM‐SUB SAL‐EC SAL‐DMN
Auditory learning delayed recall	VIS‐SM VIS‐DMN L‐CER EC‐CER DMN‐CER	SAL‐DMN DMN‐DMN SUB‐CER
Boston naming	VIS‐VIS VIS‐SAL SM‐DA DA‐SAL DA‐CER L‐DMN EC‐SUB DMN‐SUB	VIS‐DA VIS‐DMN SM‐SUB SM‐CER DA‐DMN EC‐DMN
Animal fluency	VIS‐SM VIS‐SAL VIS‐L VIS‐DMN DA‐SAL L‐DMN L‐CER	VIS‐DA VIS‐DMN SM‐DA
Clock drawing	SM‐EC EC‐SUB	VIS‐SUB SM‐SUB DA‐SUB EC‐DMN
Trail making B	VIS‐DA SM‐SUB DA‐CER SAL‐EC EC‐EC EC‐DMN DMN‐CER	SM‐SAL DA‐DA DA‐EC DMN‐DMN CER‐CER

*Note*: RSNs (e.g., DMN‐DMN) or their interactions (e.g., DMN‐EC) represented above chance in edge selection (binomial test, *α* = .05).

Abbreviations: CER, cerebellar network; DA, dorsal attention; DMN, default mode network; EC, executive control/fronto‐parietal; L, limbic; SAL, salience/ventral attention; SM, somato‐motor; SUB, subcortical; VIS, visual.

## 4 DISCUSSION

Our work provides a comprehensive whole brain and whole cognitive spectrum view on the relationship between resting‐state functional connectivity and cognition in AD and makes progress toward making subject level predictions of cognition from FC biomarkers. We accomplished that by improving the robustness of connectome predictive models of AD using If, which improved test/retest generalizability of these models and allowed for significantly improved predictions of cognition from external FC data for all seven outcomes evaluated. Finally, robust edge selection allowed for identification of RSN motifs associated with cognitive deficits in AD.

### 4.1 Differential identifiability

The use of FC as a biomarker in clinical settings requires major advancements in subject level identifiability of FC. In this work, we improve subject level FC identifiability, as measured using differential identifiability, using group level PCA. As demonstrated by other datasets (Amico & Goñi, 2018; Bari, Amico, Vike, Talavage, & Goñi, 2019), the number of PCs necessary to optimize differential identifiability corresponded to the number of subjects in the cohort (Figure 3, blue line). This indicates that while the dimensionality of the input data is twice the number of subjects (due to inclusion of test and retest data), the subject dimensionality of the data is the cutoff for a more accurate representation of individual FC, when considering small‐moderate sample sizes. Additionally, we observe a very sharp drop‐off after the peak in *I*_diff_ as was also observed by (Amico & Goñi, 2018) when If was performed on FCs generated from split time series. Finally, as shown previously (Amico & Goñi, 2018), optimizing *I*_diff_, a coarse whole brain measure (Figure 3 blue line showing *I*_diff_ and Figure S1), also robustly increased test–retest reliability at the level of individual edges (Figure S2). Optimally reconstructed FCs retained 80% of the variance in the from the original FC data (Figure 3 black dashed line), indicating that around 20% of variance present in original FC estimates is not representative of robust individual characteristics, despite the extensive preprocessing of BOLD time series used here and described in detail in (Amico et al., 2017). It is important to note that splitting an fMRI session mimics the most ideal test–retest scenario where all conditions are maintained as homogenously as possible, including scan conditions, mental state, and motion artifacts. Therefore, we would hypothesize self‐identifiability for these subjects to be very high even for original FCs reconstructed using the full range of PCs. However, the average *I*_self_ for original FCs is only 60%, in comparison to 81% for optimally reconstructed FCs. Thus, using the If framework allows us to obtain two “quality” individual FC reconstructions from the same acquisition where we previously obtained one. Finally, we observed that the optimal *I*_diff_ for this dataset is much higher than what we saw in previous data where *I*_diff_ was optimized by splitting the resting state time series in half (Amico & Goñi, 2018). We speculate that these more dramatic improvements indicate that datasets with coarse temporal acquisition or datasets including clinical populations may benefit to a greater degree from this group level PCA cleaning technique in order to improve individual level estimates of FC. However, this remains to be confirmed in additional similar datasets and across clinical diseases.

### 4.2 Effect of differential identifiability on connectome predictive modeling

When assessing clinical populations with CPM, one of the ultimate goals is to identify critical functional subcircuits associated with specific cognitive deficits. Therefore, a minimum criterion that must be met is that edge selection should be robust between test/retest data (e.g., fMRI runs or sessions) from the same subjects. Thus, we took advantage of previous splitting of fMRI data into restA and restB for purposes of uncovering connectome fingerprinting to compare edge selection performed separately on for restA versus restB FCs. Using If, we were able to improve the robustness of CPM in identifying functional subcircuits associated to specific cognitive deficits. Stability of edge selection displayed an optimal regime (12–41 PCs), after which it exponentially worsened for all outcome measures (Figure 4; see colored lines). Overlap between restA and restB edge selection (Figure 4) for optimally reconstructed data increased by an average of 30% from raw data, with an average peak overlap of 65% across outcome measures.

If did not affect the relative specificity in edge selection across outcome measures (Figure 4, black lines). Frobenius norm between outcomes remained constant around 40 and mask overlap remained constant at around 30%. This implies that the “distance” between mappings of different outcomes is preserved across PCs whereas the distance between restA‐restB mappings for a single outcome is reduced as we move from original FCs to optimally reconstructed FCs (1/2 total number of PCs). It is noteworthy that for original FCs (equivalent to reconstructing with all PCs) restA‐restB overlap approached overlap across outcomes. This implies that mappings of a single outcome based on two sessions of FCs of the same subjects are as non‐specific as the mappings of different outcomes using a single session of FC. From a clinical standpoint, where understanding which brain systems are affected is as important as predicting cognitive outcomes, this situation hampers the utility of the model. This situation is highly alleviated when performing the If prior to CPM, where restA‐restB Frobenius norm is significantly lower than across outcomes (Figure 4 left) and restA‐restB mask overlap is significantly higher than across outcomes (Figure 4 right).

In addition to improving robustness of edge selection, we also modestly improved prediction of cognitive and behavioral outcomes in new FC data from the same subjects using If. More importantly, the addition of a test/restest validation step to CPM showed that reconstructing FC at the optimal point for *I*_diff_ reduces overfitting to the training data as evidenced by a continued increase in model performance after 41 PCs for restA data from Training subjects versus a decrease after 41 PC for restB data. However, as optimally reconstructed restA and restB FCs come from the same orthogonal bases, it could be argued that their independence is further reduced upon implementation of the If, thus the improved prediction. By taking advantage of having two good quality FCs for each subject at the optimal reconstruction point, we showed that optimal reconstruction of FC significantly improved the generalization of models from the training cohorts to the validation cohorts for 5/7 the cognitive outcomes (Figure 6), although variable performance was observed across repetitions. Note that the differential identifiability pipeline was run separately on the training and validation cohorts at each repetition, thus fully maintaining the independence of training data and validation data.

We showed that splitting the timeseries prior to pre‐processing did not significantly affect the impact of If on CPM (Figure S9). Splitting the time after pre‐processing versus before pre‐processing produced similar FCs, though there was variability in subject‐wise FC similarity using the two approaches (Figure S9a). Similarity between restA and restB connectomes from the same subjects, as measured by *I*_self_, was not significantly different between the two pre‐processing approaches (Figure 3 vs. S9b). Similarly, If affected CPM equivalently for both pre‐processing approaches (Figures 4, 5, 6 vs. S9c–e). This indicates that the effects of If on CPM are robust to separate pre‐processing. However, testing of the effect of If on CPM when splitting the timeseries directly in half or when using FCs generated from two independent sessions, still needs to be directly assessed.

### 4.3 Association of resting state networks to cognitive outcomes

Previous literature making predictions from ADNI fMRI data focused solely on prediction of global cognitive status (Lin et al., 2018) or diagnostic status (Dadi et al., 2019). In contrast, we assessed the involvement of RSN systems (within and between) across cognitive deficits in AD to shed light on how FC affects cognition in AD. We found several motifs consistent with previously reported literature about the role of RSNs in AD and in general cognition. The most commonly selected networks were the DMN and the DA networks. The central role of the DMN in AD (Brier et al., 2012; Buckner et al., 2005; Garces et al., 2014; Zhou et al., 2010) and its strong associations with amyloid (Buckner et al., 2005; Hedden et al., 2009; Sperling et al., 2009; Wang et al., 2013) and tau deposition (Cope et al., 2018; Jones et al., 2016; Wang et al., 2013) has been consistently documented. The large number of tasks with significant attention components (AVLT immediate recall, Boston naming, Trail Making B) likely explains the strong involvement of interactions with the DA network being significantly overrepresented across cognitive outcomes.

Associations of within RSN interactions and cognitive function showed strong coherence to previous literature regarding the roles of RSNs in cognition and AD. We found within SAL network connectivity to be predictive of performance in immediate recall. This association is anatomically coherent as the primary role of the SAL network is in detecting salient stimuli (in this case words being spoken) and recruitment other networks to integrate these stimuli (Peters, Dunlop, & Downar, 2016). Disruption of the salience network has been found in MCI subjects in a previous study (Chand, Wu, Hajjar, & Qiu, 2017). Additionally, we found within VIS connectivity to be predictive of performance in the Boston Naming task. The involvement of the visual system in this task is obvious as it consists of confrontational word retrieval from pictures (Kaplan, Goodlass, & Weintraub, 1983). We found within network EC connectivity to be predictive of performance in MOCA and Trail Making B, both of which contain a significant executive functioning component (Arbuthnott & Frank, 2000; Nasreddine et al., 2005). The EC network is known to play a role in working memory and in the organization of goal oriented behavior (Mansouri, Rosa, & Atapour, 2015).

The association of between RSN interactions and cognitive outcomes is also coherent with previous literature and further sheds additional light on how FC alterations in AD affect cognition. We found that functional connectivity between the EC network and the SAL network was consistently associated with cognitive outcomes that included a large attention component (MOCA, AVLT immediate, Trail Making B). SAL‐EC interactions have previously been associated to performance on MOCA (Chand et al., 2017). We also identified that interactions between the VIS network and other RSNs were consistently associated with tasks that required item generation in the context of verbal memory retrieval (i.e., AVLT immediate and delayed recall, Boston Naming Test) or spontaneous generation of items belonging to a given category (i.e., animal fluency). This finding suggests an interactive role of the visual system with other functional subcircuits when executing tasks requiring semantic organization and imagery. This role of the visual system is supported by other studies identifying activation of the visual cortex and cognitive networks in imagery and semantic association tasks (Cattaneo, Vecchi, Pascual‐Leone, & Silvanto, 2009; J. Pearson, Naselaris, Holmes, & Kosslyn, 2015). Additionally, the visual cortex has also been implicated in visual short term memory and working memory (Cattaneo et al., 2009). Furthermore, in AD, connectivity of the visual system has been previously associated to neurofibrillary tangle deposition (Jones et al., 2016) and with cognitive complaints in cognitively normal or MCI subjects (Contreras et al., 2017). We identified interactions of the SM network with the EC network in the Clock Drawing task, reflecting the need for organizational planning of movement associated with the task. The involvement of these networks in the clock drawing task was also found in previous fMRI experiments using the clock drawing task on healthy aging subjects (Talwar et al., 2019). We furthermore consistently saw a significant role of cerebellar connectivity in tasks with significant motor components. Intra‐cerebellar network connectivity in Trail Making B and between network cerebellar connections were associated with performance on Trail Making B as well as MOCA (Note: the Trail Making B task is a subset of the MOCA battery) (Nasreddine et al., 2005).

Overall, findings of RSN associations consistent with previously reported roles of these RSNs in cognition and AD indicates that our unified framework not only produces robust prediction, but also produces anatomically coherent mapping of cognitive deficits to resting state functional connectivity. In depth analysis of whole brain associations between FC and cognition furthers the understanding of how changes in FC impact cognition in AD.

### 4.4 Limitations and future work

The unified identifiability‐CPM framework proposed here provides many opportunities for improving the clinical utility of FC. However, an important and necessary step to improve the clinical utility of FC is to evaluate results obtained using this unified framework on a completely external dataset such as ADNI3, which includes similar acquisition from different scanner types. This will require the estimation of final hyper parameters from the ensemble of those estimated here using the entire ADNI2 cohort and the edges appearing in the final masks. In addition to external validation of the framework, our results indicate that there are other opportunities to improve both edge selection and predictive capability of FC. Despite showing significant improvement in robustness of edge selection using our framework, we were still under 80% test/retest overlap in edge selection for all outcome measures. Edge selection may potentially be improved by taking into account the network relationship between edges, as opposed to using edgewise correlation with thresholding which treats edges as univariate independent entities. This has been previously done using methods such as partial least squares regression (Yoo et al., 2018). One could also incorporate concepts from the Network Based Statistics framework to control for spurious, small connected components (Zalesky, Fornito, & Bullmore, 2010). Controlling for such components would allow the edge selection step to be thought of as the identification of the functional subcircuits associated with a given outcome, enabling the use of network science measurements (Avena‐Koenigsberger et al., 2017; Bullmore & Sporns, 2009; Sporns, 2013) which may provide additional predictive power and provide further insight into the mechanisms of cognition and behavior. Thus, incorporating such methodologies may provide additional improvements in prediction to those shown here. As within and between network connections tend to have different properties (Noble et al., 2017), another avenue to test could be estimating separate masks and coefficients for within and between RSN connections. Finally, CPM may also prove useful in predicting change in cognitive outcomes over time (Pena‐Nogales et al., 2018), thus assessing the effect of differential identifiability on connectome predictive of longitudinal outcomes in AD would be a worthy contribution toward improving FC utility as a clinical biomarker.

## 5 CONCLUSIONS

Our framework improved the robustness of individual level prediction of cognition from FC, which is the first step toward clinical use of FC and better understanding of how functional connectivity supports cognition in AD. We showed that the joint framework of differential identifiability with connectome predictive modeling improves the quality of models obtained from CPM in terms of stability of edge selection, test/retest generalizability, and generalizability to external data. Additionally, we showed that the use of two FC sessions from each subject provides a unique perspective when assessing and validating connectome predictive models. Finally, improving the robustness of edge selection allowed for reliable assessment of the associations between functional connectivity and cognitive deficits in AD. Our findings indicate both specific and global associations of resting state functional connectivity with cognitive deficits in AD which are consistent with previous literature regarding the roles resting state networks play in both cognition and AD.

## CONFLICT OF INTEREST

The authors of this manuscript declare no conflicts of interest.

## Supporting information


**Figure S1** Average Identifiability matrices over the 1,000 repetitions for original FCs and FCs reconstructed at the optimal point for differential identifiability. (Left) Average matrices in the training cohorts. (Right) Average Matrices for the validation cohorts.

**Figure S2** Average edgewise ICC over 1,000 repetitions for original FCs and FCs reconstructed at the optimal point for differential identifiability. Edges in ICC matrices are ordered according to RSN membership. (**left**) Edgewise ICC matrix for original FCs. (**middle**) Edgewise ICC matrix for optimally reconstructed FCs. (**right**) Distribution of edgewise ICC values before for original FCs (blue, 82 PCs) and optimally reconstructed FCs (red, 41 PCs).

**Figure S3** Over represented edges (binomial test, α = 0.01) for the Montreal Cognitive Association Test (MOCA). Positively associated edges (**left**) and negatively associated edges (**right**) are visualized separately. Nodes are sized according to their degree and colored according to resting state network membership. Positive mask edges are colored blue while negative mask edges are colored red.

**Figure S4** Over represented edges (binomial test, α = 0.01) for the AVLT Immediate Recall test. Positively associated edges (l**eft**) and negatively associated edges (**right**) are visualized separately. Nodes are sized according to their degree and colored according to resting state network membership. Positive mask edges are colored blue while negative mask edges are colored red.

**Figure S5** Over represented edges (binomial test, α = 0.01) for the AVLT Delayed Recall test. Positively associated edges (**left**) and negatively associated edges **(right**) are visualized separately. Nodes are sized according to their degree and colored according to resting state network membership. Positive mask edges are colored blue while negative mask edges are colored red.

**Figure S6** Over represented edges (binomial test, α = 0.01) for the Boston Naming Test. Positively associated edges (**left**) and negatively associated edges (**right**) are visualized separately. Nodes are sized according to their degree and colored according to resting state network membership. Positive mask edges are colored blue while negative mask edges are colored red.

**Figure S7** Over represented edges (binomial test, α = 0.01) for the Clock Drawing Test. Positively associated edges (**left**) and negatively associated edges (**right**) are visualized separately. Nodes are sized according to their degree and colored according to resting state network membership. Positive mask edges are colored blue while negative mask edges are colored red.

**Figure S8** Over represented edges (binomial test, α = 0.01) for the Trail Making B test. Positively associated edges (**left**) and negatively associated edges (**right**) are visualized separately. Nodes are sized according to their degree and colored according to resting state network membership. Positive mask edges are colored blue while negative mask edges are colored red.

**Figure S9** Ι*f*‐CPM workflow performed on FCs split in an interleaved fashion (as if TR = 6 s) prior to pre‐processing. **(A)** Pairwise correlation of averaged restA‐restB FCs with corresponding FCs generated when split was performed after pre‐processing. **(B)** Average behavior Ι*f* on the validation cohort. **(C)** (Colored Lines) Frobenius norm of correlation matrices associated to each outcome measure for restA FCs versus restB FCs. (Black line) Average pairwise Frobenius Norm of correlation matrices between two different outcome measures using only restA FCs. **(D)** (Colored Lines) Mask overlap between restA FCs versus RestB FCs, for each outcome measure. (Black Line) Average pairwise mask overlap between two different outcomes using only restA FCs. **(E)** Correlation between estimated and expected outcomes from models fit using restA FCs. **(F)** Correlation between estimated and expected outcomes when models fit on restA Training FCs were applied to restB FCs from the same subjects.

## Data Availability

Code and example data associated to this article will be uploaded to the CONNplexity lab website publications page. https://engineering.purdue.edu/ConnplexityLab/publications
