# Supplementary figures and images for: Optimizing differential identifiability improves connectome predictive modeling of cognitive deficits from functional connectivity in Alzheimer's disease
Source: Hum Brain Mapp. 2021 May 5;42(11):3500–16. doi: 10.1002/hbm.25448 (PMC8249900; doi:10.1002/hbm.25448)

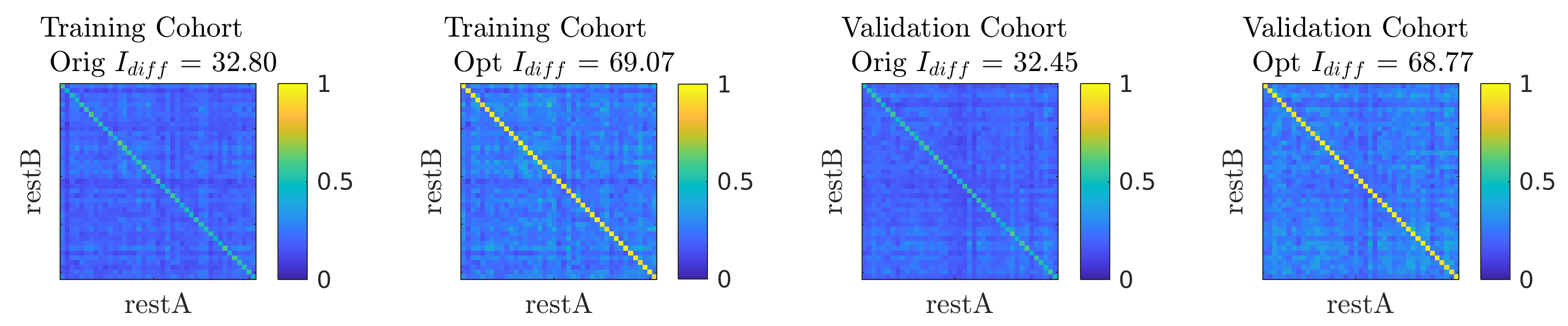

Supplement: Supplementary file 1 — Figure S1 Average Identifiability matrices over the 1,000 repetitions for original FCs and FCs reconstructed at the optimal point for differential identifiability. (Left) Average matrices in the training cohorts. (Right) Average Matrices for the validation cohorts. [file HBM-42-3500-s004.png]

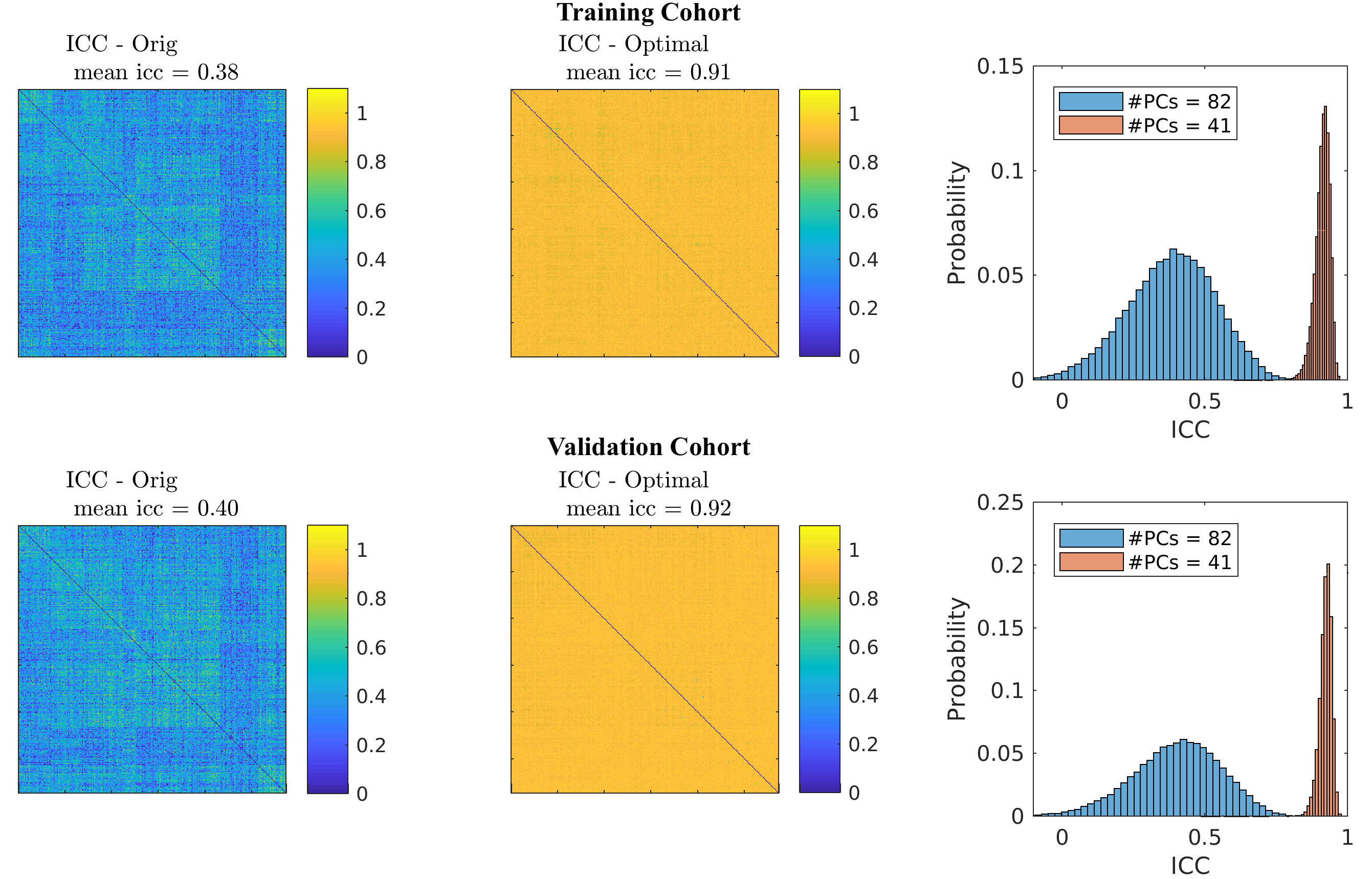

Supplement: Supplementary file 2 — Figure S2 Average edgewise ICC over 1,000 repetitions for original FCs and FCs reconstructed at the optimal point for differential identifiability. Edges in ICC matrices are ordered according to RSN membership. (left) Edgewise ICC matrix for original FCs. (middle) Edgewise ICC matrix for optimally reconstructed FCs. (right) Distribution of edgewise ICC values before for original FCs (blue, 82 PCs) and optimally reconstructed FCs (red, 41 PCs). [file HBM-42-3500-s005.png]

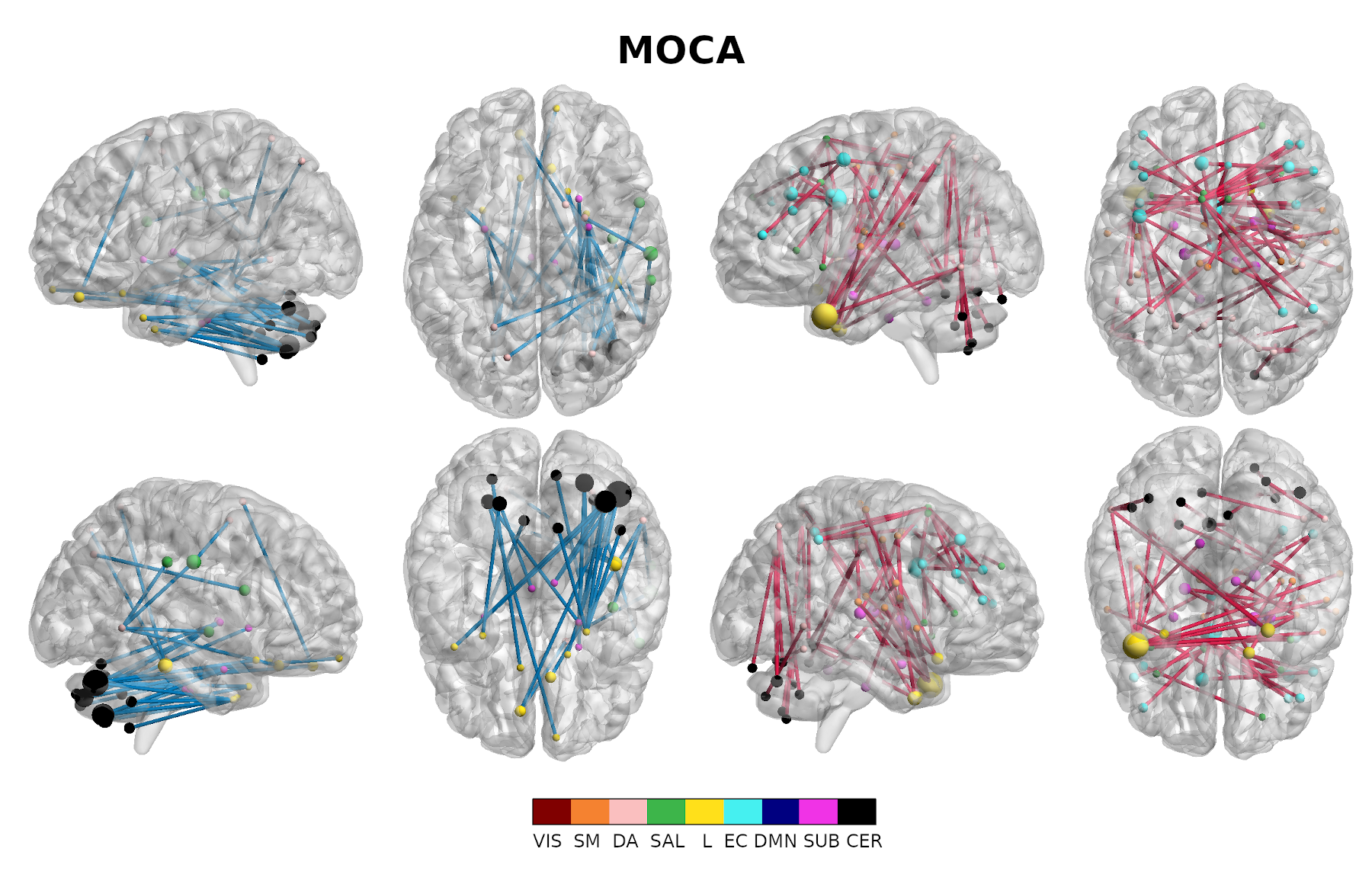

Supplement: Supplementary file 3 — Figure S3 Over represented edges (binomial test, α = 0.01) for the Montreal Cognitive Association Test (MOCA). Positively associated edges (left) and negatively associated edges (right) are visualized separately. Nodes are sized according to their degree and colored according to resting state network membership. Positive mask edges are colored blue while negative mask edges are colored red. [file HBM-42-3500-s007.png]

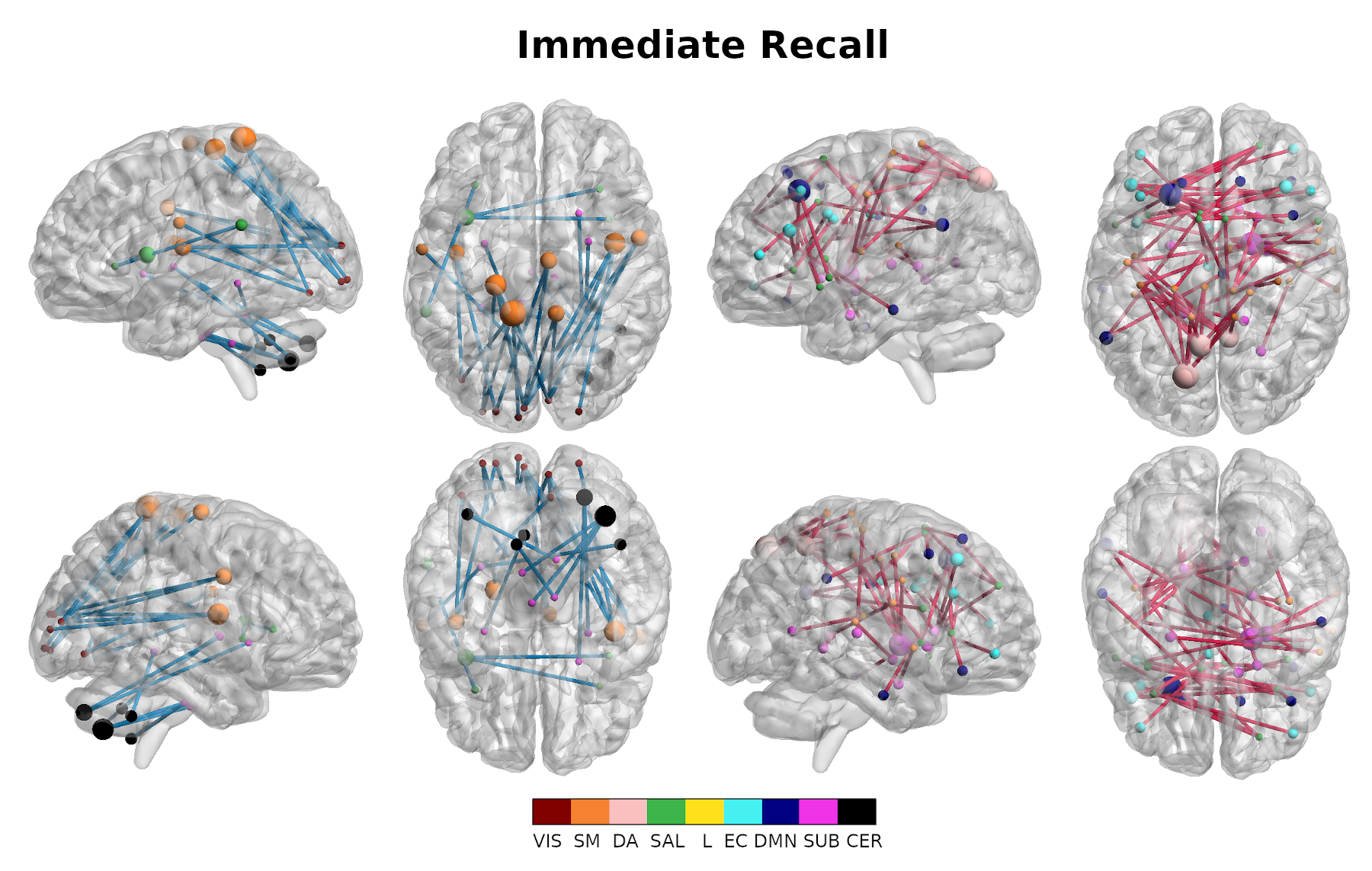

Supplement: Supplementary file 4 — Figure S4 Over represented edges (binomial test, α = 0.01) for the AVLT Immediate Recall test. Positively associated edges (left) and negatively associated edges (right) are visualized separately. Nodes are sized according to their degree and colored according to resting state network membership. Positive mask edges are colored blue while negative mask edges are colored red. [file HBM-42-3500-s006.png]

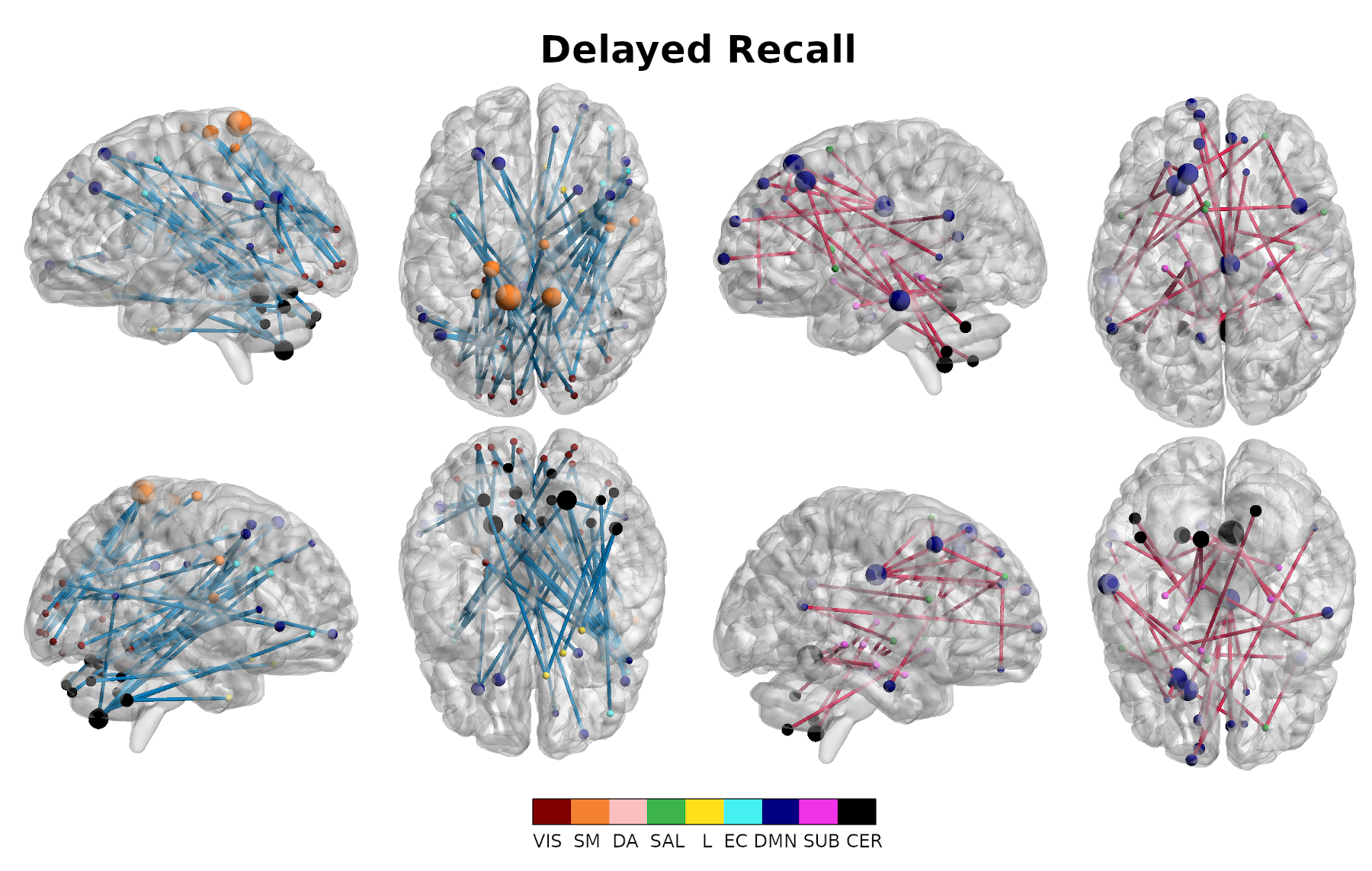

Supplement: Supplementary file 5 — Figure S5 Over represented edges (binomial test, α = 0.01) for the AVLT Delayed Recall test. Positively associated edges (left) and negatively associated edges (right) are visualized separately. Nodes are sized according to their degree and colored according to resting state network membership. Positive mask edges are colored blue while negative mask edges are colored red. [file HBM-42-3500-s002.png]

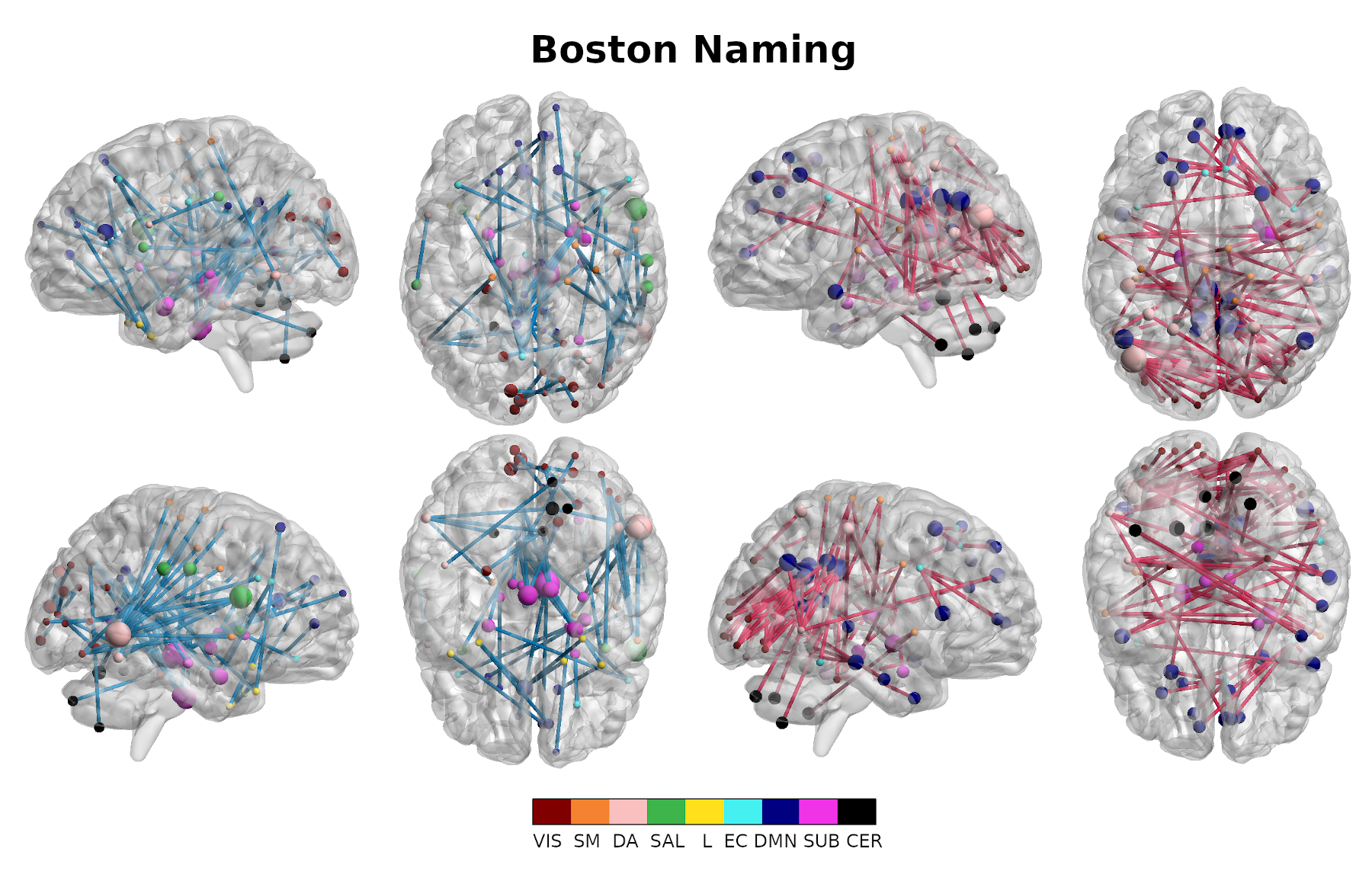

Supplement: Supplementary file 6 — Figure S6 Over represented edges (binomial test, α = 0.01) for the Boston Naming Test. Positively associated edges (left) and negatively associated edges (right) are visualized separately. Nodes are sized according to their degree and colored according to resting state network membership. Positive mask edges are colored blue while negative mask edges are colored red. [file HBM-42-3500-s001.png]

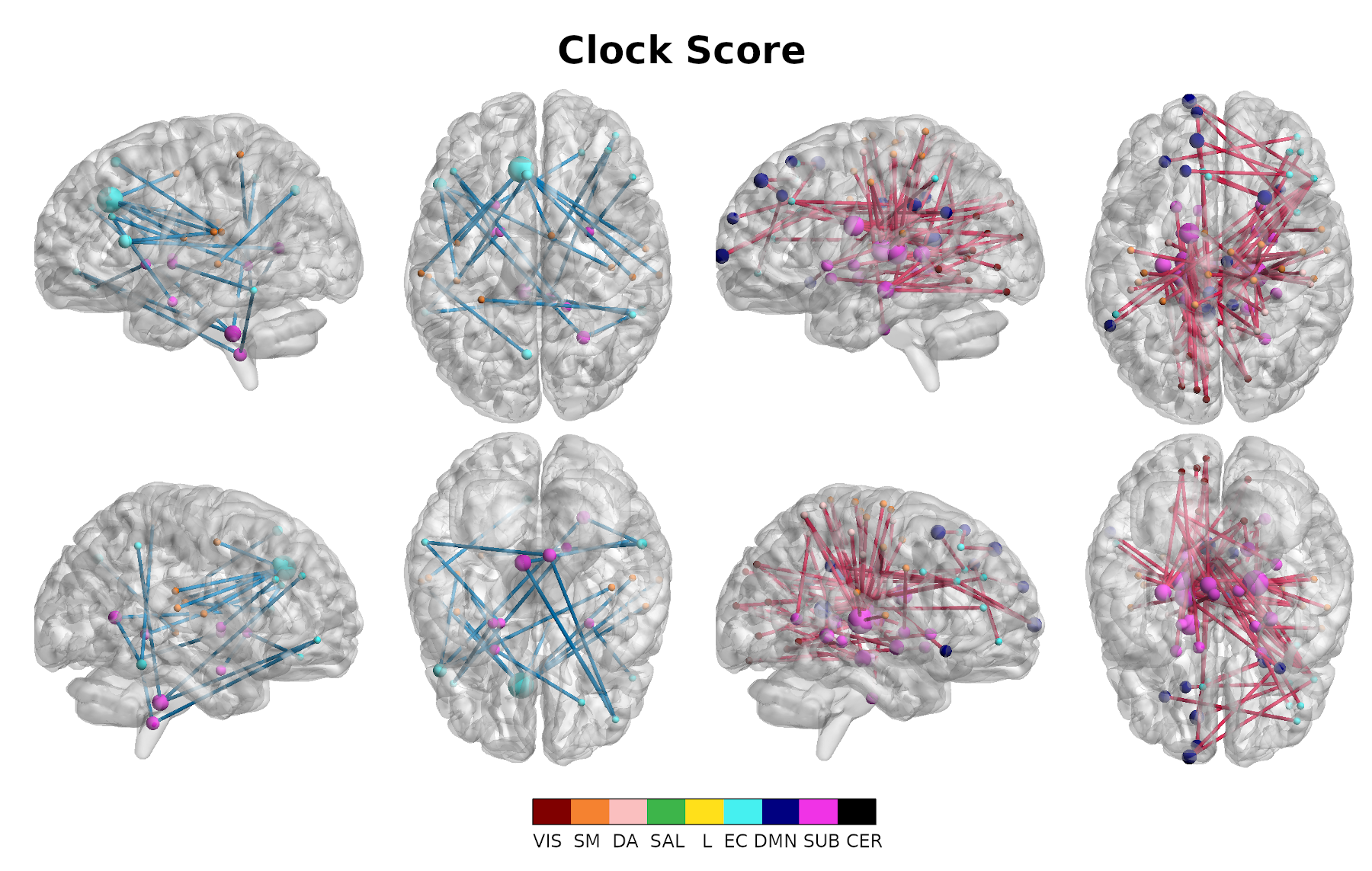

Supplement: Supplementary file 7 — Figure S7 Over represented edges (binomial test, α = 0.01) for the Clock Drawing Test. Positively associated edges (left) and negatively associated edges (right) are visualized separately. Nodes are sized according to their degree and colored according to resting state network membership. Positive mask edges are colored blue while negative mask edges are colored red. [file HBM-42-3500-s008.png]

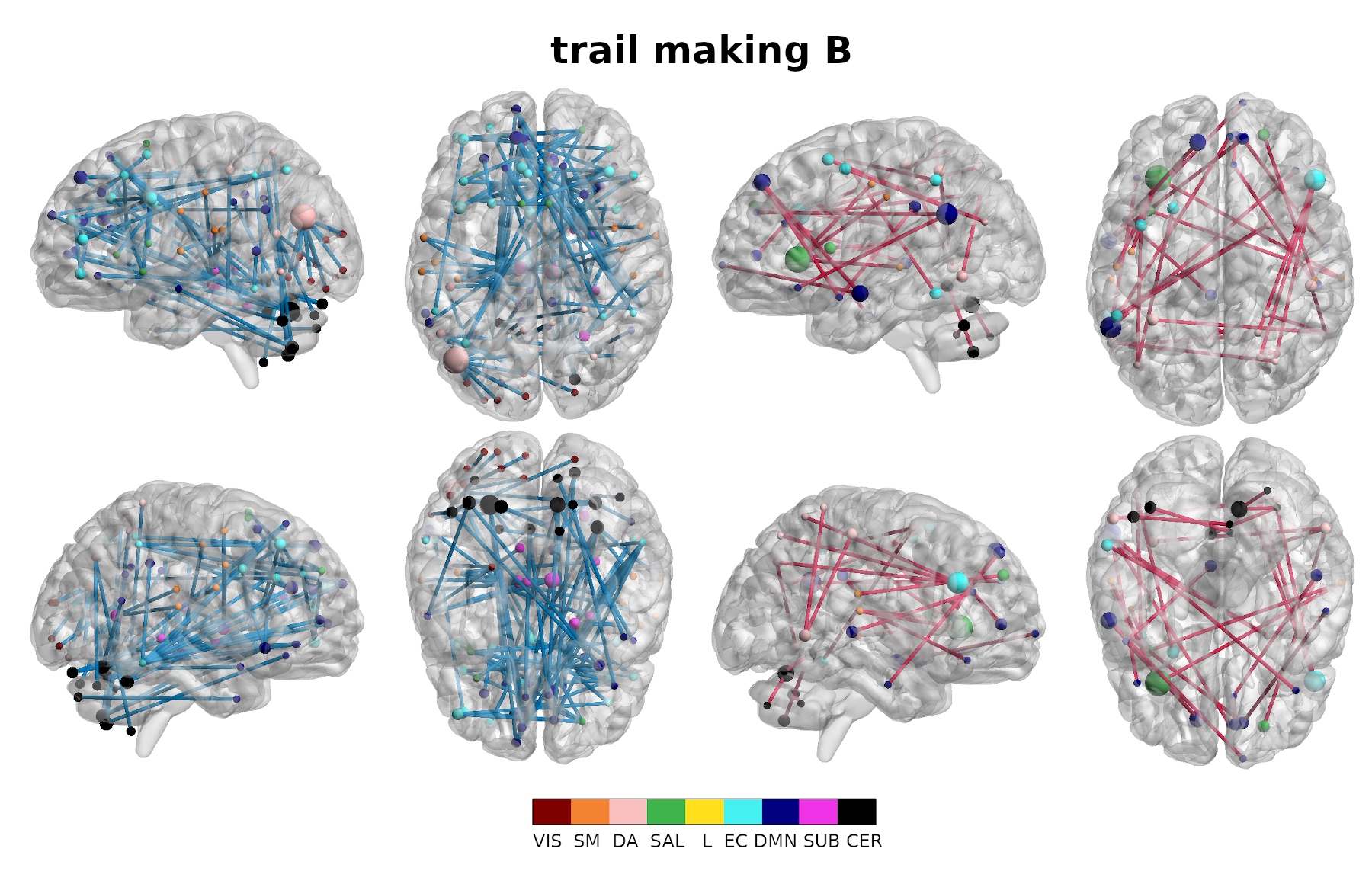

Supplement: Supplementary file 8 — Figure S8 Over represented edges (binomial test, α = 0.01) for the Trail Making B test. Positively associated edges (left) and negatively associated edges (right) are visualized separately. Nodes are sized according to their degree and colored according to resting state network membership. Positive mask edges are colored blue while negative mask edges are colored red. [file HBM-42-3500-s003.png]

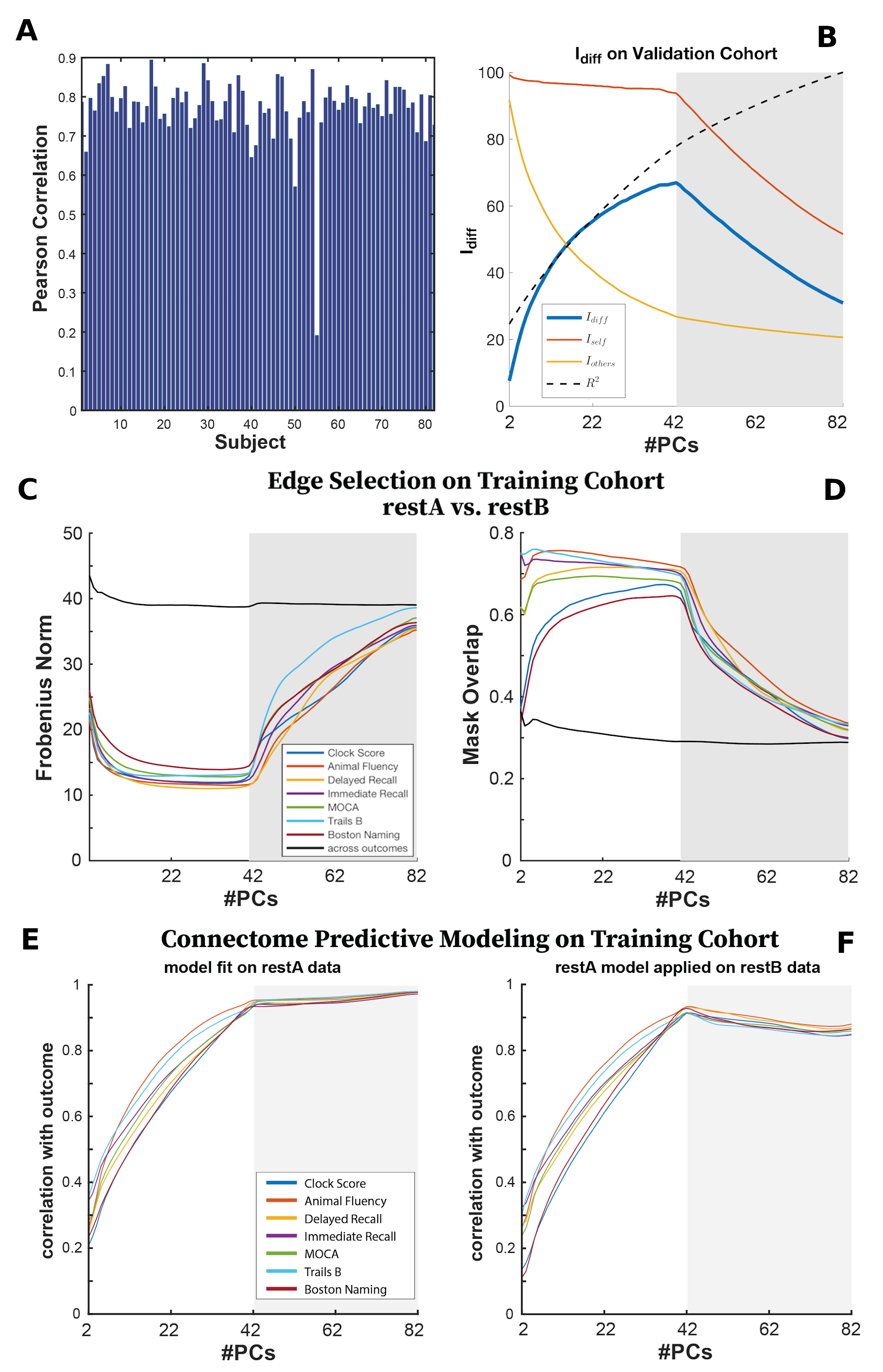

Supplement: Supplementary file 9 — Figure S9 Ιf‐CPM workflow performed on FCs split in an interleaved fashion (as if TR = 6 s) prior to pre‐processing. (A) Pairwise correlation of averaged restA‐restB FCs with corresponding FCs generated when split was performed after pre‐processing. (B) Average behavior Ιf on the validation cohort. (C) (Colored Lines) Frobenius norm of correlation matrices associated to each outcome measure for restA FCs versus restB FCs. (Black line) Average pairwise Frobenius Norm of correlation matrices between two different outcome measures using only restA FCs. (D) (Colored Lines) Mask overlap between restA FCs versus RestB FCs, for each outcome measure. (Black Line) Average pairwise mask overlap between two different outcomes using only restA FCs. (E) Correlation between estimated and expected outcomes from models fit using restA FCs. (F) Correlation between estimated and expected outcomes when models fit on restA Training FCs were applied to restB FCs from the same subjects. [file HBM-42-3500-s009.png]
